# Differential synthesis of novel small protein times *Salmonella* virulence program

**DOI:** 10.1371/journal.pgen.1010074

**Published:** 2022-03-04

**Authors:** Hubert Salvail, Jeongjoon Choi, Eduardo A. Groisman

**Affiliations:** 1 Department of Microbial Pathogenesis, Yale School of Medicine, New Haven, Connecticut, United States of America; 2 Department of Molecular, Cellular and Developmental Biology, Yale University, New Haven, Connecticut, United States of America; 3 Department of Genetics, Yale School of Medicine, New Haven, Connecticut, United States of America; 4 Yale Microbial Sciences Institute, West Haven, Connecticut, United States of America; Michigan State University, UNITED STATES

## Abstract

Gene organization in operons enables concerted transcription of functionally related genes and efficient control of cellular processes. Typically, an operon is transcribed as a polycistronic mRNA that is translated into corresponding proteins. Here, we identify a bicistronic operon transcribed as two mRNAs, yet only one allows translation of both genes. We establish that the novel gene *ugtS* forms an operon with virulence gene *ugtL*, an activator of the master virulence regulatory system PhoP/PhoQ in *Salmonella enterica* serovar Typhimurium. Only the longer *ugtSugtL* mRNA carries the *ugtS* ribosome binding site and therefore allows *ugtS* translation. Inside macrophages, the *ugtSugtL* mRNA species allowing translation of both genes is produced hours before that allowing translation solely of *ugtL*. The small protein UgtS controls the kinetics of PhoP phosphorylation by antagonizing UgtL activity, preventing premature activation of a critical virulence program. Moreover, *S*. enterica serovars that infect cold-blooded animals lack *ugtS*. Our results establish how foreign gene control of ancestral regulators enables pathogens to time their virulence programs.

## Author summary

Pathogens must express their virulence genes at precisely the right time to cause disease. Here, we identify a novel small protein that governs a critical virulence program in the pathogen *Salmonella enterica* serovar Typhimurium (*S*. Typhimurium). We establish that the novel small protein UgtS prevents the virulence protein UgtL from activating the master virulence regulator PhoP inside macrophages. *S*. Typhimurium produces two *ugtSugtL* mRNAs, but only one of them allows *ugtS* translation. The absence of *ugtS* from *S*. *enterica* serovars that infect cold-blooded animals raises the possibility of UgtS playing a regulatory role during infection of warm-blooded animals. Our findings establish how a horizontally acquired bicistron enables pathogens to time their virulence programs by controlling ancestral regulators.

## Introduction

**Fig 1:**
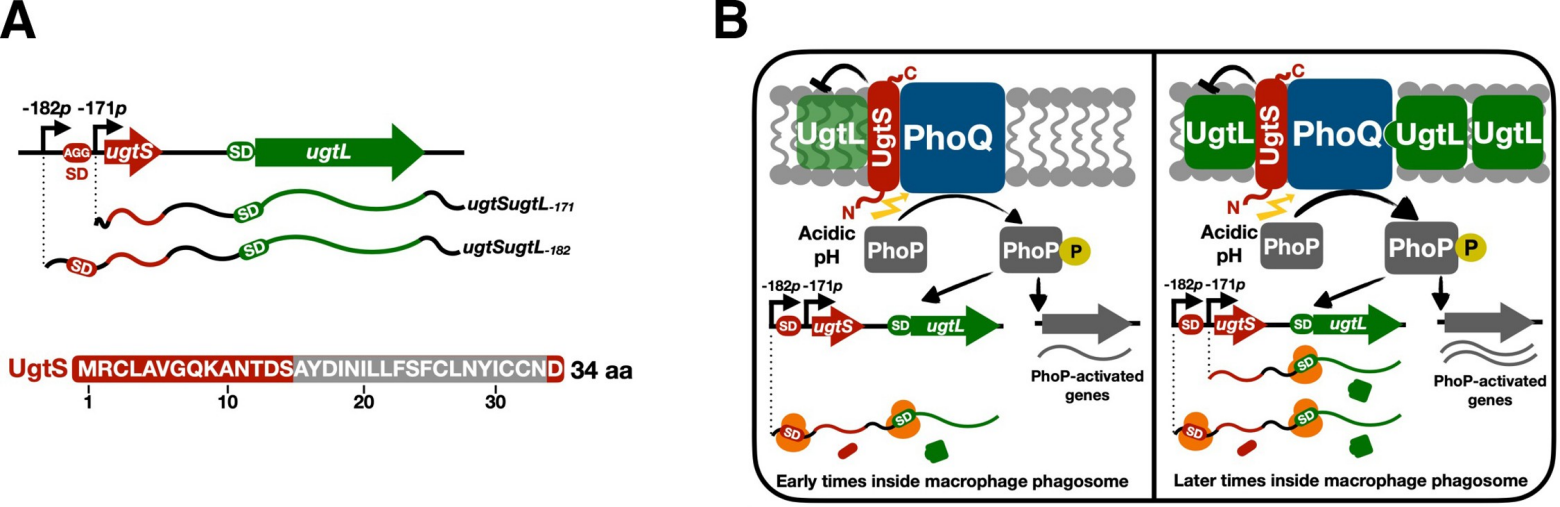
Two different mRNAs from the *ugtSugtL* bicistron time the *S*. Typhimurium PhoP virulence program inside macrophages. (A, *Top*) Genetic map of the *ugtSugtL* chromosomal region. The *ugtS* gene was previously annotated as *STM14_1939*. The -182*p* and -171*p* transcription start sites (TSSs) are indicated by arrows. The *ugtS* Shine-Dalgarno (SD) sequence (AGG) is boxed in red. (A, *Bottom*) Deduced amino acid sequence for the *ugtS* gene. aa, amino acids. The predicted transmembrane domain (predicted by TMpred [78]) is boxed in gray. (B, *left*) At early times following *S*. Typhimurium internalization by macrophages, mildly acidic pH promotes PhoQ autophosphorylation, which then phosphorylates the response regulator PhoP. Phosphorylated PhoP (PhoP-P) activates *ugtSugtL* transcription from the -182*p* TSS, resulting in synthesis of both UgtS and UgtL, the latter being in insufficient amounts to overcome antagonization by UgtS and fully promote PhoQ autophosphorylation, and then activation of PhoP and of its regulon. (B, *right*) At later times following bacterial internalization by macrophages, PhoP activates *ugtSugtL* transcription from both the -182*p* and the -171*p* TSSs, resulting in the synthesis of UgtS and increased amounts of the UgtL protein, enhancing PhoQ autophosphorylation, and then PhoP activation, leading to increased transcription of PhoP-activated genes. UgtS antagonizes UgtL, thereby delaying the full activation of the PhoP regulon until later times inside macrophages. This delay may result from the simultaneous interaction of UgtS with the UgtL and PhoQ proteins. UgtS is depicted as an inner membrane protein with the N-terminus in the cytoplasm and the C-terminus in the periplasm (N_in_-C_out_), as predicted by TMpred [78].

Organisms respond to a change in conditions by modifying the repertoire of expressed gene products. In bacteria, operons enable the joint transcription of genes specifying products that are part of the same biochemical pathway or that mediate the response to a particular signal [1,2]. Usually, this results in a single polycistronic mRNA that is translated into corresponding proteins [3–5]. Here, we report a singular example of a bicistronic operon transcribed as two mRNAs, only one of which allows translation of both genes (Fig 1A). This differential synthesis of a small protein by transcript isoforms controls a virulence program of the facultative intracellular pathogen *Salmonella enterica* serovar Typhimurium (*S*. Typhimurium) operating inside macrophages (Fig 1B).

*S*. Typhimurium virulence is governed by the PhoP/PhoQ two-component system [6–9]. The sensor PhoQ responds to specific signals by promoting the phosphorylated, active state of the regulatory protein PhoP (PhoP-P), which binds specific DNA sequences and changes transcription of the corresponding genes, including those required for survival inside macrophages [10]. PhoQ activation by mildly acidic pH is critical for *S*. Typhimurium virulence because inhibition of phagosome acidification impairs both PhoP activation [11,12] and bacterial survival inside macrophages [13] and also because *S*. Typhimurium mutants defective in PhoQ activation by mildly acidic pH are attenuated for virulence [14].

The PhoP-activated *Salmonella*-specific *ugtL* gene is necessary for PhoQ activation in mildly acidic pH and therefore for *S*. Typhimurium virulence [15]. UgtL is an inner membrane protein that increases the PhoP-P-to-PhoP ratio by enhancing PhoQ autophosphorylation [15]. Curiously, two transcription start sites have been mapped for the *ugtL* gene (Fig 1A), resulting in two *ugtL* mRNAs that differ in 11 nt. These mRNAs have unusually long 5’ leader regions of 182 and 171 nt in length [16], suggesting that *ugtL* expression is subjected to additional regulatory inputs via the 5’ leader region. Moreover, this raises the question about the functional significance of having two *ugtL* mRNAs with similarly long 5’ leader regions.

We now report that the *ugtL* gene forms an operon with the novel gene *ugtS* (currently annotated as *STM14_1939;* Fig 1A). We establish that *ugtS* specifies a small protein that binds to both the UgtL and PhoQ proteins, thereby hindering PhoP activation. We determine that the two mRNAs produced from the *ugtSugtL* bicistron differ in that the longer mRNA results in translation of both genes, whereas the shorter mRNA permits translation of *ugtL* only because it lacks the *ugtS* ribosome binding site. Inside macrophages, *S*. Typhimurium delays activation of the PhoP/PhoQ virulence program by producing the longer *ugtSugtL* mRNA hours before the shorter *ugtSugtL* mRNA (Fig 1B). Absent from *S*. *enterica* serovars that infect cold-blooded animals, the *ugtS* gene may provide the means to time a virulence program in warm-blooded hosts.

## Results

### Only one of the two mRNAs produced from the *ugtSugtL* bicistron allows *ugtS* translation

The two *ugtL* transcripts include a 34-sense-codon-long open reading frame (ORF; annotated as *STM14_1939* and herein named *ugtS*) starting 164 nt upstream of the *ugtL* start codon [17] (Fig 1A). Curiously, the predicted ribosome binding site for *ugtS* (AGG) is present only in the longer of the two *ugtSugtL* mRNAs (Fig 1A), suggesting that the longer (*ugtSugtL*_*-182*_) transcript allows *ugtS* translation but the shorter (*ugtSugtL*_*-171*_) one does not.

**Fig 2:**
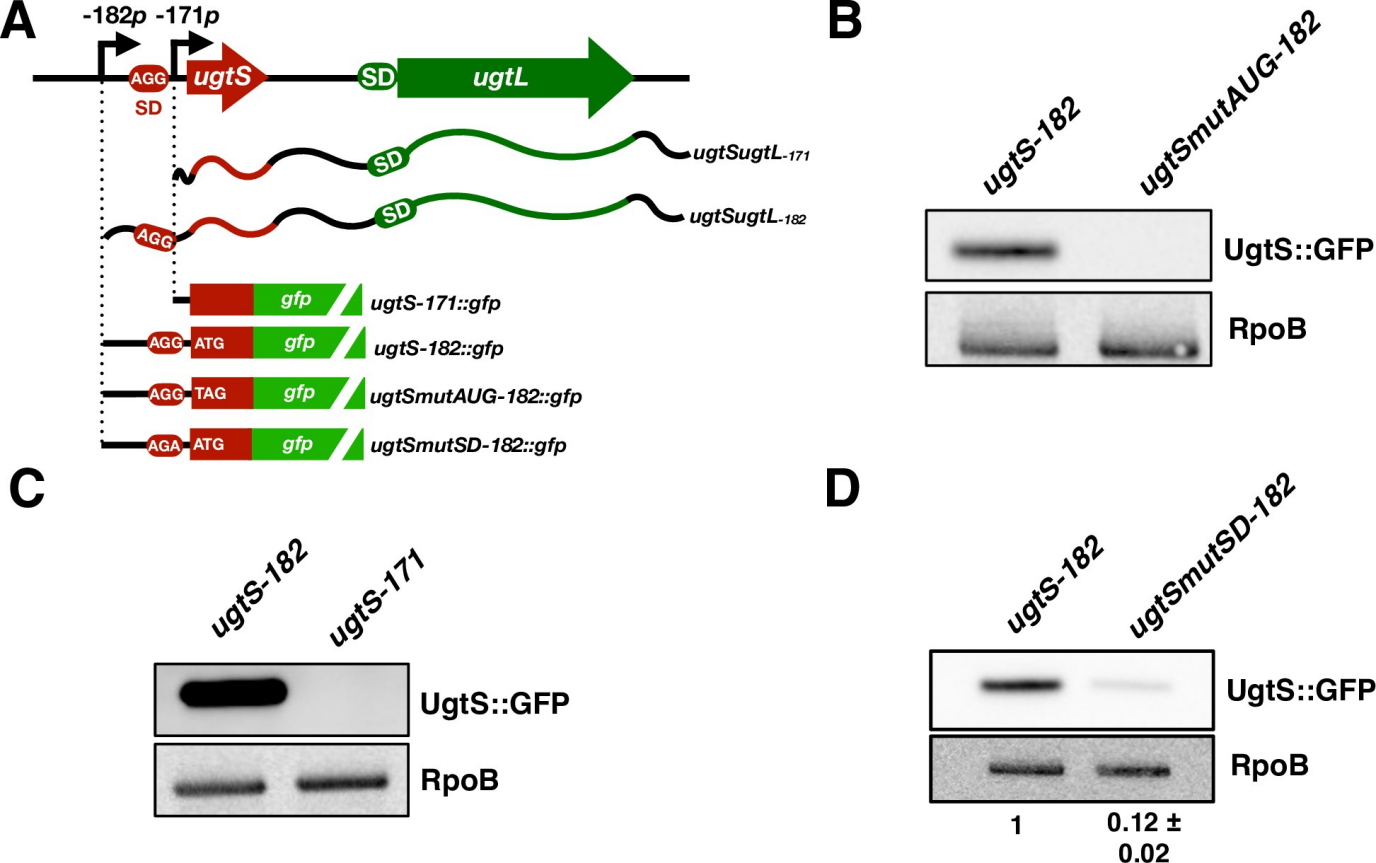
Only the longer of the two mRNAs produced from the *ugtSugtL* bicistron allows *ugtS* translation. (A) Schematic of the *ugtSugtL* chromosomal region and of the *ugtS* and *ugtL* GFP fusions used in the experiments described in Fig 2B, 2C and 2D. SD, Shine-Dalgarno sequence (boxed in red and green for *ugtS* and *ugtL*, respectively). The -182*p* and -171*p* (positions relative to *ugtL* ATG start codon) transcription start sites are indicated with arrows. (B) Western blot analysis of crude extracts from wild-type *S*. Typhimurium harboring pXG10sf-*ugtS-182* or pXG10sf-*ugtSmutAUG-182* grown in N-minimal acidic pH (pH 4.9, 1 mM MgCl_2_) media for 6 h (late log phase). Samples were analyzed with antibodies directed to the GFP or RpoB proteins. Data are representative of two independent experiments, which gave similar results. (C) Western blot analysis of crude extracts from wild-type *S*. Typhimurium harboring pXG10sf-*ugtS-182* or pXG10sf-*ugtS-171* grown in N-minimal acidic pH (pH 4.9, 1 mM MgCl_2_) media for 6 h (late log phase). Samples were analyzed with antibodies directed to the GFP or RpoB proteins. Data are representative of two independent experiments, which gave similar results. (D) Western blot analysis of crude extracts from wild-type *S*. Typhimurium harboring pXG10sf-*ugtS-182* or pXG10sf-*ugtSmutSD-182* grown in N-minimal acidic pH (pH 4.9, 1 mM MgCl_2_) media for 6 h (late log phase). Samples were analyzed with antibodies directed to the GFP or RpoB proteins. Numbers below blot indicate UgtS::GFP amounts for the pXG10sf-*ugtSmutSD-182-*carrying strain relative to pXG10sf-*ugtS-182-*carrying strain. Data are representative of two independent experiments, which gave similar results.

We established that the *ugtS* gene is translated because Western blot analysis of crude extracts from wild-type *S*. Typhimurium harboring a low copy number plasmid with a translational fusion of *ugtS* (region -182 to -63 relative to the *ugtL* start codon) to the *gfp* gene under the control of a constitutive promoter (*ugtS-182*::*gfp*) (Fig 2A) showed a band of the predicted UgtS-GFP size following growth in mildly acidic pH (Fig 2B). By contrast, wild-type *S*. Typhimurium harboring an isogenic plasmid in which the *ugtS* start codon was replaced by a stop codon (Fig 2A) did not show the UgtS-GFP band (Fig 2B). These data are in agreement with ribosome profiling experiments showing ribosome occupancy of the *ugtS* ORF [18].

We determined that *ugtS* translation requires the ribosome binding site present in the 11 nt that distinguish the two *ugtSugtL* transcripts (Fig 2A). That is, UgtS-GFP was produced by wild-type *S*. Typhimurium harboring the *ugtS-182*::*gfp* construct in which *ugtS*::*gfp* is constitutively transcribed from the -182*p* transcription start site (Fig 2C) but absent from the isogenic strain with the *ugtS-171*::*gfp* construct in which *ugtS*::*gfp* is constitutively transcribed from the -171*p* transcription start site (Fig 2C). In support of this notion, UgtS-GFP amounts were eight-fold lower in wild-type *S*. Typhimurium harboring the *ugtSmutSD-182*::*gfp* derivative with a single nucleotide substitution in the *ugtS* Shine-Dalgarno sequence (Fig 2A) than in the isogenic strain with the wild-type sequence (Fig 2D). Taken together, these results establish that only the longer of the two *ugtSugtL* mRNAs allows *ugtS* translation.

The data presented in this section raise the question: What is the physiological significance of producing two *ugtSugtL* transcripts that differ in their ability to synthesize the small protein UgtS?

### The two *ugtSugtL* mRNAs exhibit distinct expression behaviors in mildly acidic pH

**Fig 3:**
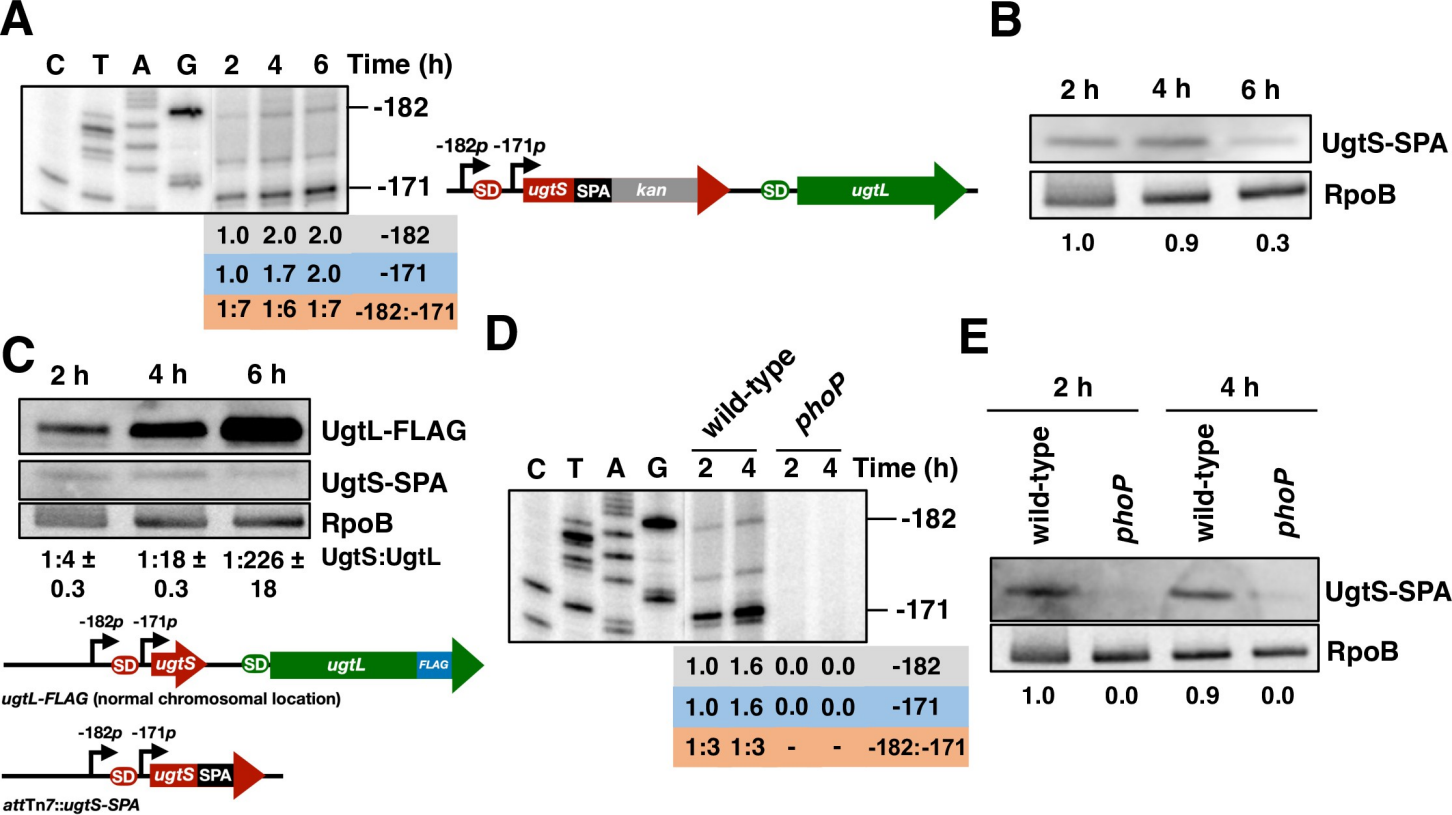
The *ugtSugtL*_*-182*_ and *ugtSugtL*_*-171*_ transcripts are PhoP-activated and exhibit distinct expression patterns in mildly acidic pH, resulting in differential abundance of the UgtS and UgtL proteins. (A) Primer extension analysis of the *ugtSugtL*_*-182*_ and *ugtSugtL*_*-171*_ transcripts in *ugtS-SPA* (HS1170) *S*. Typhimurium grown in N-minimal acidic pH medium (pH 4.9, 1 mM MgCl_2_) for the indicated times. Primer extension was carried out on total RNA using primer W4055. The band observed at position -176 relative to the *ugtL* start codon corresponds to a W4055 primer-specific reverse transcription artefact. Transcript abundance relative to the leftmost lane is indicated for the -182 and -171 transcripts. -182:-171 transcript abundance ratios are indicated below the blot. The *ugtS-SPA*::*Km*^*R*^ construct is depicted in the right panel. The -182*p* and -171*p* (positions relative to *ugtL* ATG start codon) transcription start sites are indicated with arrows. SD, Shine-Dalgarno sequences (red and green for *ugtS* and *ugtL*, respectively). Data are representative of two independent experiments, which gave similar results. (B) Western blot analysis of crude extracts from *ugtS-SPA* (HS1170) *S*. Typhimurium grown in N-minimal acidic pH medium (pH 4.9, 1 mM MgCl_2_) for the indicated times. Samples were analyzed with antibodies directed to the FLAG epitope or the RpoB protein. Protein abundance relative to the leftmost lane is indicated below the blot. Data are representative of two independent experiments, which gave similar results. (C) Western blot analysis of crude extracts from *att*Tn*7*::*ugtS-SPA ugtL-FLAG* (HS1940) *S*. Typhimurium grown in N-minimal acidic pH medium (pH 4.9, 1 mM MgCl_2_) for the indicated time points. Samples were analyzed with antibodies directed to the FLAG epitope or the RpoB protein. UgtL/UgtS protein amounts ratios are marked below. Data are representative of two independent experiments, which gave similar results. The *att*Tn*7*::*ugtS-SPA ugtL-FLAG* construct is depicted in the lower panel. The -182*p* and -171*p* (positions relative to *ugtL* ATG start codon) transcription start sites are indicated with arrows. SD, Shine-Dalgarno sequences (red color for *ugtS* Shine-Dalgarno sequence, green color for *ugtL* Shine-Dalgarno sequence). (D) Primer extension analysis of *ugtSugtL*_*-182*_ and *ugtSugtL*_*-171*_ transcripts levels in *ugtS-SPA* (HS1170) and *ugtS-SPA phoP* (HS1178) *S*. Typhimurium grown in N-minimal acidic pH medium (pH 4.9, 1 mM MgCl_2_) for the indicated time points. Primer extension reaction was carried out on total RNA samples using primer W4055. The band observed at position -176 relative to the *ugtL* start codon corresponds to a W4055 primer-specific reverse transcription artefact. Transcript abundance relative to the leftmost lane is indicated for the -182 and -171 transcripts. -182:-171 transcript abundance ratios are marked below. Data are representative of two independent experiments, which gave similar results. (E) Western blot analysis of crude extracts from *att*Tn*7*::*ugtS-SPA* (HS1795) and *att*Tn*7*::*ugtS-SPA phoP* (HS1823) *S*. Typhimurium grown in N-minimal acidic pH medium (pH 4.9, 1 mM MgCl_2_) for the indicated time points. Samples were analyzed with antibodies directed to the FLAG epitope or RpoB protein. Protein abundance relative to the leftmost lane is marked below. Data are representative of two independent experiments, which gave similar results.

We examined the production of the two *ugtSugtL* mRNAs in a *S*. Typhimurium strain expressing a UgtS-SPA protein from the normal *ugtS* chromosomal location and promoter and harboring a *Km*^*R*^ cassette downstream (Fig 3A). This strain enabled us to determine whether there is a correlation between the production of the two *ugtSugtL* transcripts and the synthesis of the UgtS protein. Bacteria were grown in defined media with mildly acidic pH, a condition that activates PhoQ in a *ugtL*-dependent manner [15]. The abundance of the *ugtSugtL*_*-171*_ transcript increased over 6 h (Fig 3A), whereas the amount of the *ugtSugtL*_*-182*_ transcript was lower than that of the *ugtSugtL*_*-171*_ transcript and did not change between 4 and 6 h (Fig 3A). Curiously, UgtS-SPA abundance was lower at 6 h than at 2 and 4 h (Fig 3B), possibly resulting from increased protein turnover at 6 h. Therefore, UgtS-SPA abundance is inversely correlated with abundance of the *ugtSugtL*_*-171*_ transcript.

To determine how changes in the abundance of the two transcripts impact the amounts of the UgtS and UgtL proteins, we engineered a strain specifying a UgtL-FLAG protein from its normal chromosomal location and harboring a 460-nt region from the *ugtSugtL* promoter and leader regions specifying a UgtS-SPA protein at the *att*Tn*7* site (Fig 3C). This strategy, which has been successfully used to examine the regulation of other PhoP-regulated genes [19], enables the study of gene regulation with the relevant promoter elements in single copy in the chromosome and was necessary because antibodies to the native UgtS and UgtL proteins are not available. UgtS-SPA abundance in the engineered strain decreased between 4 and 6 h in mildly acidic pH (Fig 3C), recapitulating the behavior of the strain specifying UgtS-SPA from the normal chromosomal location (Fig 3B). Conversely, UgtL abundance increased dramatically between 2 and 6 h (Fig 3C), in parallel with the increase in the *ugtSugtL*_*-171*_ transcript (Fig 3A). Thus, the UgtS-to-UgtL protein ratio went from 1: 4 at 2 h, to 1: 18 at 4 h, and 1: 226 at 6 h (Fig 3C), increasing 56-fold in a 4 h period.

Production of both *ugtSugtL*_*-182*_ and *ugtSugtL*_*-171*_ mRNAs is PhoP-dependent because the transcripts were absent from an isogenic *phoP* null mutant strain (Fig 3D). The UgtS protein bearing a C-terminal SPA tag was detected at 2 and 4 h in mildly acidic pH conditions in the wild-type strain but not in the *phoP* mutant (Fig 3E). Collectively, these data demonstrate that transcription of the *ugtSugtL* bicistron and production of the UgtS protein are PhoP-dependent in bacteria experiencing mildly acidic pH. The two mRNAs exhibit different abundances that, in turn, give rise to different amounts of the UgtS and UgtL proteins.

### UgtS reduces PhoP phosphorylation by decreasing UgtL activity

Operons typically specify proteins that participate in the same pathway, albeit sometimes in opposite directions. For example, certain operons specify both a toxin and an antitoxin that counteracts the effects of the toxin [20]. Similarly, the PhoP-activated *mgtCBRU* operon specifies the virulence proteins MgtC and MgtB as well as the 30-residue-long MgtR, which promotes degradation of both MgtC [21] and MgtB [22], and the 28-residue-long MgtU, which prevents proteolysis of MgtB but not MgtC [22]. Thus, we considered the possibility of the 34-residue-long UgtS controlling UgtL amounts, which, in turn, would impact the PhoP-P-to-PhoP ratio.

**Fig 4:**
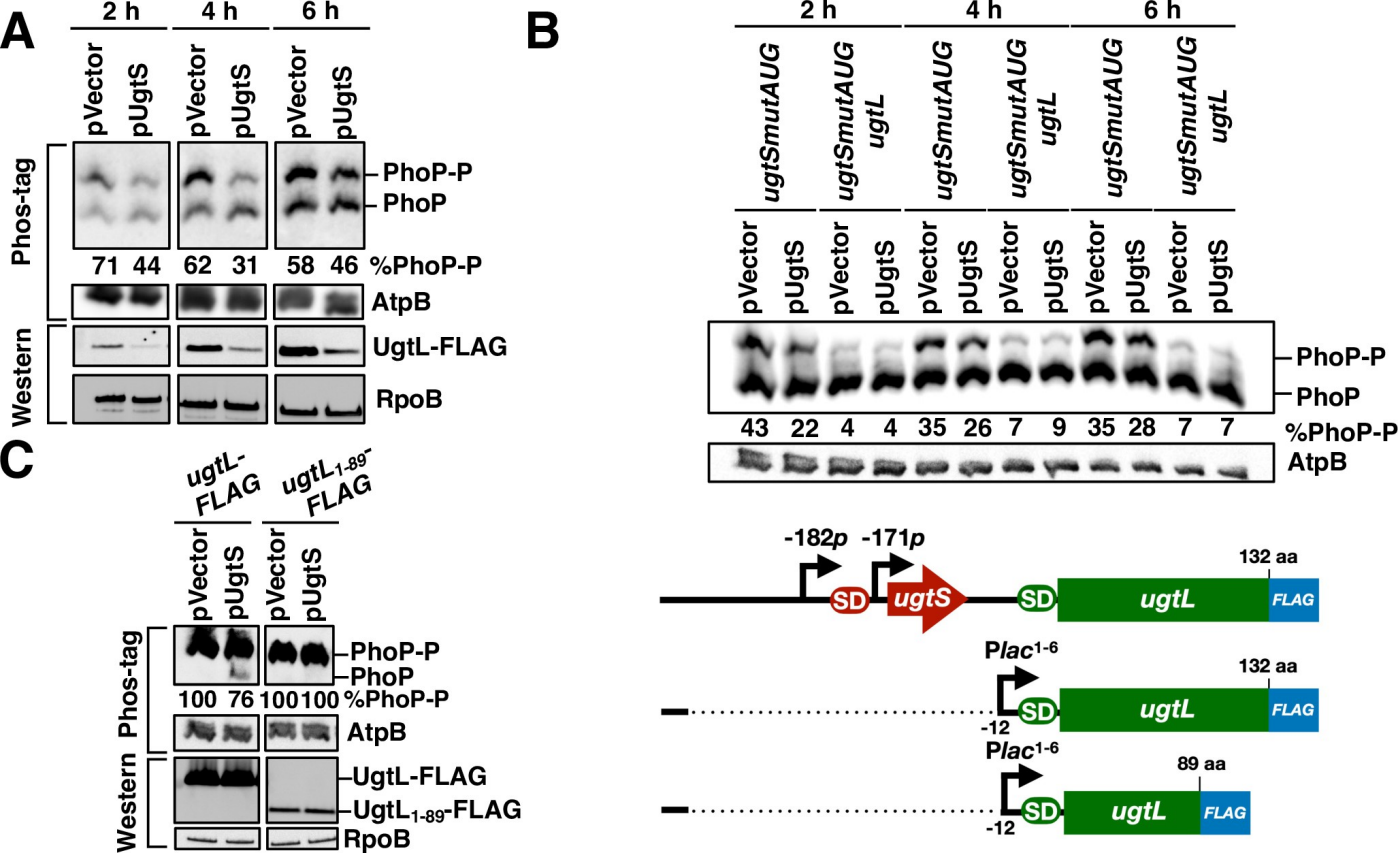
UgtS reduces PhoP activity. (A) Western blot analysis of extracts prepared from *ugtL-FLAG ugtSmutAUG* (HS1207) *S*. Typhimurium harboring plasmid or pVector (empty pUHE-21 vector) grown for the indicated time points in N-minimal acidic pH medium (pH 4.9, 1 mM MgCl_2_) supplemented with 0.2 mM IPTG before inoculation. Samples run on Phos-tag gels (upper panel) were analyzed with antibodies directed to the PhoP and AtpB proteins, and those run on regular SDS-PAGE (lower panel) were analyzed with antibodies directed to the FLAG epitope or the RpoB protein. Numbers under the blots indicate the percentage of phosphorylated PhoP (PhoP-P). Data are representative of two independent experiments, which gave similar results. (B) Western blot analysis of extracts prepared from *ugtSmutAUG* (HS1119) and *ugtL ugtSmutAUG* (HS1548) *S*. Typhimurium harboring plasmid pUgtS or pVector (empty pUHE-21 vector) grown and analyzed as in (A). Data are representative of three independent experiments, which gave similar results. (C) Western blot analysis of extracts prepared from P*lac1-6_-12ugtL-FLAG* (JC1362) and P*lac1-6_-12ugtL*_*1-89*_*-FLAG* (JC1414) *S*. Typhimurium harboring plasmid pUgtS or pVector (empty pUHE-21 vector) grown for 6 h (late log phase) in N-minimal acidic pH medium (pH 4.9, 1 mM MgCl_2_) supplemented with 0.2 mM IPTG before inoculation. Blots were analyzed as in (A). Data are representative of three independent experiments, which gave similar results. The p*lac1-6_-12ugtL-FLAG* and p*lac1-6_-12ugtL*_*1-89*_*-FLAG* constructs are depicted in the right panel. SD, Shine-Dalgarno sequences red and green for *ugtS* and *ugtL*, respectively), aa, amino acids, -12 refers to position -12 relative to the *ugtL* start codon.

A chromosomal *ugtSmutAUG* mutant with the *ugtS* start codon replaced by a stop codon had lower UgtL protein amounts when harboring a plasmid with the *ugtS* gene transcribed from a heterologous promoter than with the vector control (Fig 4A). That *ugtS* expression from a heterologous promoter decreases UgtL abundance produced from the normal chromosomal location and promoter (Fig 4A) indicates that UgtS reduces UgtL abundance in *trans* and argues against *ugtS* controlling UgtL abundance by a transcription attenuation-like mechanism [23].

The PhoP-P-to-PhoP ratio was lower in the *ugtS-*expressing strain than in the vector control (Fig 4A), in agreement with UgtS decreasing UgtL amounts (Fig 4A) and UgtL being necessary to promote PhoP phosphorylation in mildly acidic pH [15]. UgtS’s ability to reduce the PhoP-P-to-PhoP ratio is strictly dependent on UgtL because the *ugtS*-expressing plasmid did not decrease the PhoP-P-to-PhoP ratio in a *ugtSmutAUG ugtL* double mutant (Fig 4B) but did so in the isogenic *ugtSmutAUG* single mutant (Fig 4B). The decrease in PhoP-P-to-PhoP ratio resulting from heterologous *ugtS* expression was more pronounced at 2 and 4 h than at 6 h (Fig 4A and 4B), suggesting that UgtS operates in a time-dependent manner.

UgtS may reduce UgtL amounts by stimulating UgtL degradation and/or by hindering UgtL’s ability to activate PhoQ [15], which would reduce transcription of the PhoP-activated targets, including the *ugtSugtL* operon. To test these possibilities and avoid the confounding effects of PhoP and UgtL positively activating one another [15,24], we engineered a strain with a chromosomal *ugtL-FLAG* gene transcribed from the PhoP-independent p*lac*_*1-6*_ promoter [25,26] and lacking *ugtS* because it is missing 170 nt starting 12 nt upstream of the *ugtL* start codon (Fig 4C) (the *ugtL-FLAG* gene specifies a functional UgtL protein [15]). We then examined the PhoP-P-to PhoP ratio and UgtL abundance in isogenic strains carrying either the *ugtS*-expressing plasmid or the vector control. The PhoP-P-to-PhoP ratio was lower in the strain with the *ugtS*-expressing plasmid than in that with the vector control (Fig 4C, left two lanes). By contrast, similar amounts of C-terminally FLAG-tagged UgtL were present in the two strains (Fig 4C). These results establish that UgtS reduces UgtL activity (i.e., promoting PhoP-P) rather than UgtL amounts.

### UgtS interacts with the UgtL and PhoQ proteins

We reasoned that UgtS antagonizes UgtL through direct interaction with UgtL and/or PhoQ because: (i) UgtL binds to PhoQ and increases its autophosphorylation activity [15]; (ii) phosphorylated PhoQ is the only known PhoP phosphodonor and only PhoP-P phosphatase [27]; (iii) UgtS reduces the PhoP-P-to-PhoP ratio in a *ugtL*-dependent manner (Fig 4B); and (iv) UgtS is predicted to localize to the inner membrane, like the PhoQ [28] and UgtL [15] proteins.

**Fig 5:**
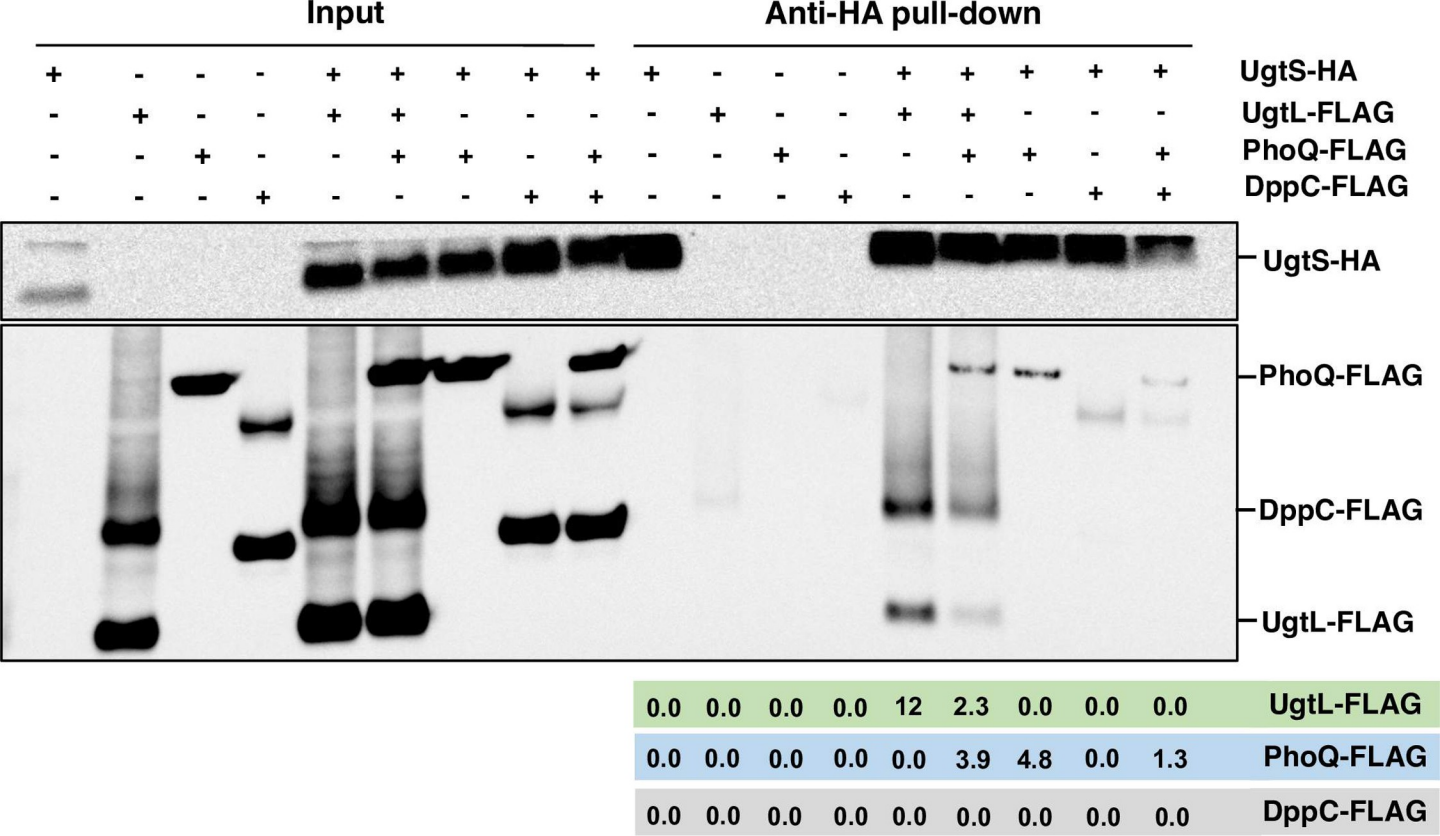
UgtS interacts with the UgtL and PhoQ proteins. Anti-HA pull-down assay showing interactions between *in vitro*–synthesized UgtS-HA, UgtL-FLAG, PhoQ-FLAG, and DppC-FLAG proteins. Samples were analyzed by Western blotting using antibodies recognizing the HA or FLAG epitopes. Numbers under the anti-HA pull-down portion of the blot indicate the protein amounts in arbitrary units of the pulled-down FLAG-tagged proteins. Data are representative of three independent experiments, which gave similar results.

We established that UgtS interacts with both UgtL and PhoQ because immunoprecipitation experiments with *in vitro* synthesized UgtS-HA, UgtL-FLAG, and PhoQ-FLAG proteins demonstrated that UgtL-FLAG and PhoQ-FLAG are pulled down by anti-HA antibodies (Fig 5). By contrast, the inner membrane protein DppC-FLAG used as negative control was not (Fig 5). When the experiment was carried out with the UgtS-HA, UgtL-FLAG, and PhoQ-FLAG proteins in the same tube, lower amounts of UgtL-FLAG were pulled down than when the reaction was carried out in the absence of PhoQ-FLAG (Fig 5). These data suggest that UgtS and UgtL binding to PhoQ are mutually exclusive and that UgtS binds PhoQ with higher affinity than UgtL.

### UgtS-dependent reduction in the PhoP-P-to-PhoP ratio requires UgtL’s C-terminal residues but is independent of PhoQ antagonist MgrB

A derivative of the UgtL protein missing the 33 C-terminal amino acids retains the ability to activate the sensor PhoQ [15]. By contrast, UgtL’s 33 C-terminal amino acids are required for UgtS to reduce the PhoP-P-to-PhoP ratio under acidic pH conditions. This is because the UgtS-expressing plasmid decreased the PhoP-P-to-PhoP ratio in a *ugtL*^*+*^ strain but not in one expressing the UgtL protein missing the 33 C-terminal amino acids (Fig 4C).

MgrB is a PhoP-activated small protein that inhibits PhoQ’s ability to promote PhoP phosphorylation directly [29,30]. Though UgtS binds to PhoQ (Fig 5) and reduces the PhoP-P-to-PhoP ratio (Fig 4), it does so independently of MgrB because heterologous transcription of the *ugtS* gene decreased both UgtL protein amounts and the PhoP-P-to-PhoP ratio in a *ugtSmutAUG mgrB* mutant strain (S1 Fig). The PhoP-P-to-PhoP ratio was higher in the *ugtSmutAUG mgrB* double mutant than in the *ugtSmutAUG* single mutant (S1 Fig), indicating that MgrB operates in a *ugtS*-independent manner and in agreement with UgtL promoting PhoP phosphorylation in an *mgrB*-independent manner [15]. Thus, the UgtS-mediated reduction of PhoP-P amounts is UgtL-dependent and MgrB-independent.

### UgtS controls the kinetics of PhoP activation in mildly acidic pH and inside macrophages

**Fig 6:**
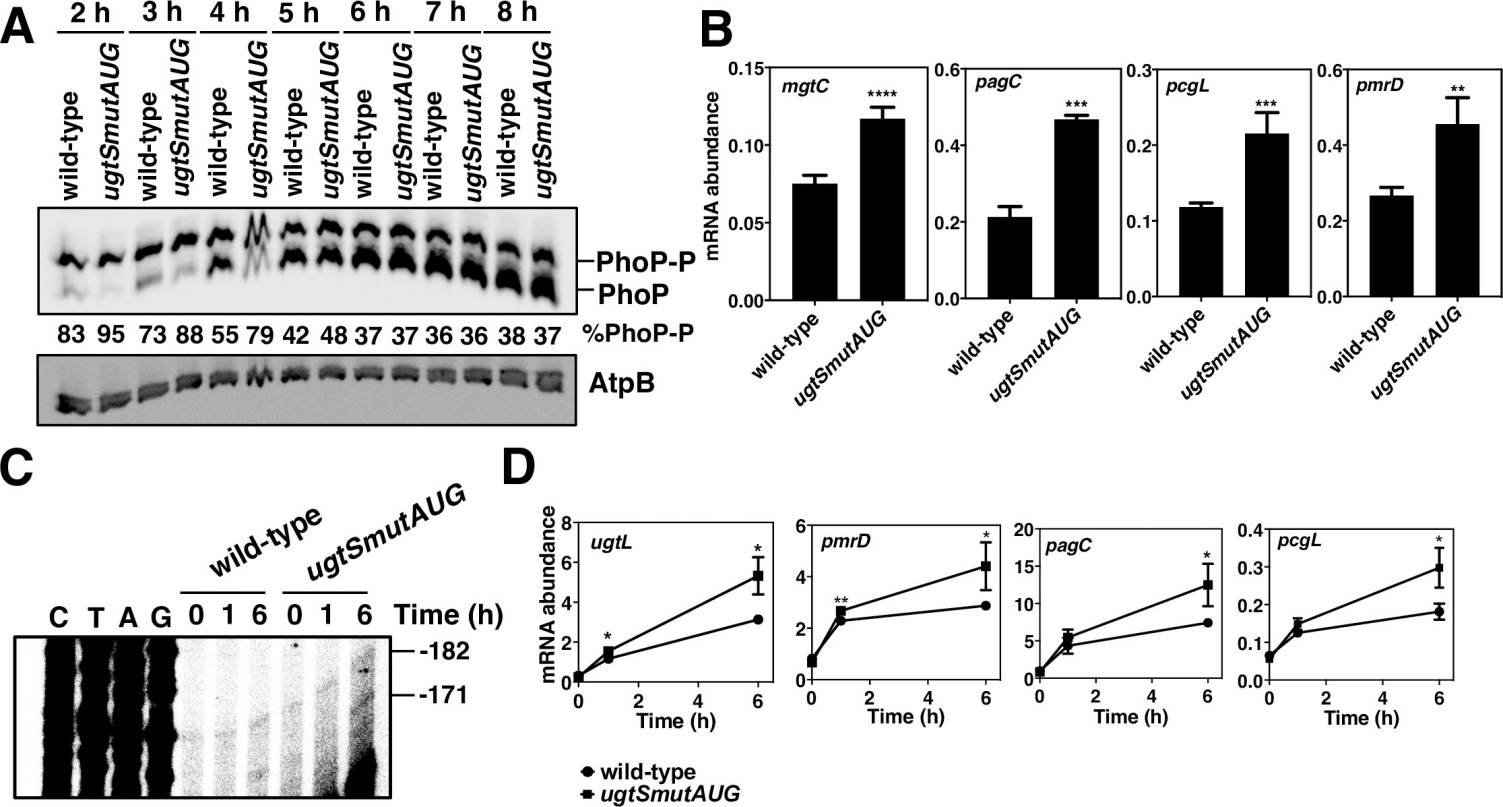
UgtS modulates the kinetics of PhoP activation in acidic pH conditions and inside macrophages. (A) Western blot analysis of extracts prepared from wild-type (14028s) and *ugtSmutAUG* (HS1119) *S*. Typhimurium grown in N-minimal acidic pH medium (pH 4.9, 1 mM MgCl_2_) for the indicated times. Samples were run on Phos-tag gels analyzed with antibodies directed to the PhoP and AtpB proteins. Numbers under the blots indicate the percentage of phosphorylated PhoP (PhoP-P). Data are representative of three independent experiments, which gave similar results. (B) Abundance of *mgtC*, *pagC*, *pcgL*, and *pmrD* transcripts in wild-type (14028s) and *ugtSmutAUG* (HS1119) *S*. Typhimurium grown in N-minimal acidic pH medium (pH 4.9, 1 mM MgCl_2_) for 4 h (mid-log phase). Transcript abundance values were normalized to the abundance of 16S ribosomal RNA. The mean and SD from at least three independent experiments are shown. **P < 0.01, ***P < 0.001, ****P < 0.0001, two-tailed unpaired *t* test with *ugtSmutAUG* vs. wild type. (C) Primer extension analysis of *ugtSugtL*_*-182*_ and *ugtSugtL*_*-171*_ transcripts from wild-type (14028s) and *ugtSmutAUG* (HS1119) *S*. Typhimurium harvested from J774A.1 macrophages at the indicated times. Primer extension reaction was carried out on total RNA samples using primer W4469. A gel image with lower exposure is provided as supplementary data (S3 Fig) to better discern the DNA sequencing ladder bands. Data are representative of two independent experiments, which gave similar results. (D) Abundance of *ugtL*, *pmrD*, *pagC*, and *pcgL* transcripts in wild-type (14028s) and *ugtSmutAUG* (HS1119) *S*. Typhimurium inside J774A.1 macrophages at the indicated times. Transcript abundance values were normalized to the abundance of 16S ribosomal RNA. The mean and SD from three independent experiments are shown. *P < 0.05, **P < 0.01, two-tailed unpaired *t* test with *ugtSmutAUG* vs. wild type. The absence of a * indicates no significant difference between mutant and wild type.

We determined that the PhoP-P-to-PhoP ratio is higher in the *ugtSmutAUG* mutant than in wild-type *S*. Typhimurium at 2, 3, and 4 h in mildly acidic pH (Fig 6A). However, the ratio was the same for both strains at 5, 6, 7, and 8 h in the same medium (Fig 6A). These results are consistent with UgtS amounts peaking between 2 and 4 h in mildly acidic pH (Fig 3C) and with UgtS expression decreasing the PhoP-P-to-PhoP ratio to a greater extent at 2 and 4 h than at 6 h (Fig 4A). The mRNA abundance of the PhoP-activated genes *mgtC*, *pagC*, *pcgL*, and *pmrD* was higher in the *ugtSmutAUG* mutant than in wild-type *S*. Typhimurium at 4 h in mildly acidic pH (Fig 6B), reflecting that the PhoP-P-to-PhoP ratio is higher in the former than in the latter strain (Fig 6A) and that PhoP-P is the form of the PhoP protein that activates transcription of these genes [10].

We reasoned that UgtS should impact the kinetics of PhoP activation during infection because UgtS reduces PhoP activity in mildly acidic pH, which *S*. Typhimurium experiences inside macrophage phagosomes and results in PhoP activation [11,13]. To test this hypothesis, we examined the mRNA abundance of PhoP-activated genes and the profile of the *ugtSugtL*_*-182*_ and *ugtSugtL*_*-171*_ transcripts at different times after *S*. Typhimurium internalization by macrophages.

At 1 h post infection of the macrophage-like cell line J774.1, the *ugtSugtL*_*-182*_ transcript was detected, but the *ugtSugtL*_*-171*_ transcript was not (Fig 6C). At this time, the mRNA abundance was similar between wild-type and *ugtSmutAUG* mutant strains for the PhoP-activated *pagC* and *pcgL* genes (Fig 6D) and slightly higher for the PhoP-activated *ugtL* and *pmrD* genes in the *ugtSmutAUG* strain as compared to the wild-type strain (Fig 6D). Apparently, the *ugtL* mRNA produced by 1 h post internalization is insufficient to activate PhoP (Fig 6C and 6D and [31]). This is because only the *ugtSugtL*_*-182*_ transcript participates in the total UgtL synthesis output at this time (Fig 6C), which is probably insufficient in providing enough UgtL protein to enhance PhoQ activity and overcome antagonization by UgtS (Fig 6D). This is also consistent with the *ugtSmutAUG* mutation not impacting much PhoP activation at this time point (Fig 6D).

At 6 h post infection, the *ugtSugtL*_*-171*_ transcript was detected (Fig 6C), and its presence was accompanied by an increase in mRNA abundance of the *ugtL*, *pmrD*, *pagC*, and *pcgL* genes in wild-type *S*. Typhimurium (Fig 6D). Thus, enough *ugtL* has been expressed (and UgtL accumulated) to activate PhoP at this time (Fig 6C and 6D) [31]. The mRNA abundance of the investigated PhoP-activated genes (Fig 6D) was higher in the *ugtSmutAUG* mutant than in the wild-type strain, in agreement with UgtS decreasing the PhoP-P-to-PhoP ratio in a UgtL-dependent manner (Fig 4B) and UgtL activating PhoP [31].

Taken together, the results presented in this section indicate that *ugtSugtL* mRNA isoforms differing in both the time of production and ability to produce the UgtS protein control the kinetics with which PhoP-activated genes are expressed in macrophages. PhoP activation does not require UgtL at early times, when *S*. Typhimurium produces the *ugtSugtL*_*-182*_ mRNA, a transcript allowing production of both UgtS and UgtL. By contrast, the *ugtSugtL*_*-171*_ mRNA produced at later times enables translation of *ugtL* but not *ugtS*, resulting in PhoP activation by UgtL and negative feedback by UgtS.

### The *ugtS* gene is narrowly distributed in non-typhoidal *S*. *enterica* serovars that infect warm-blooded animals

**Fig 7:**
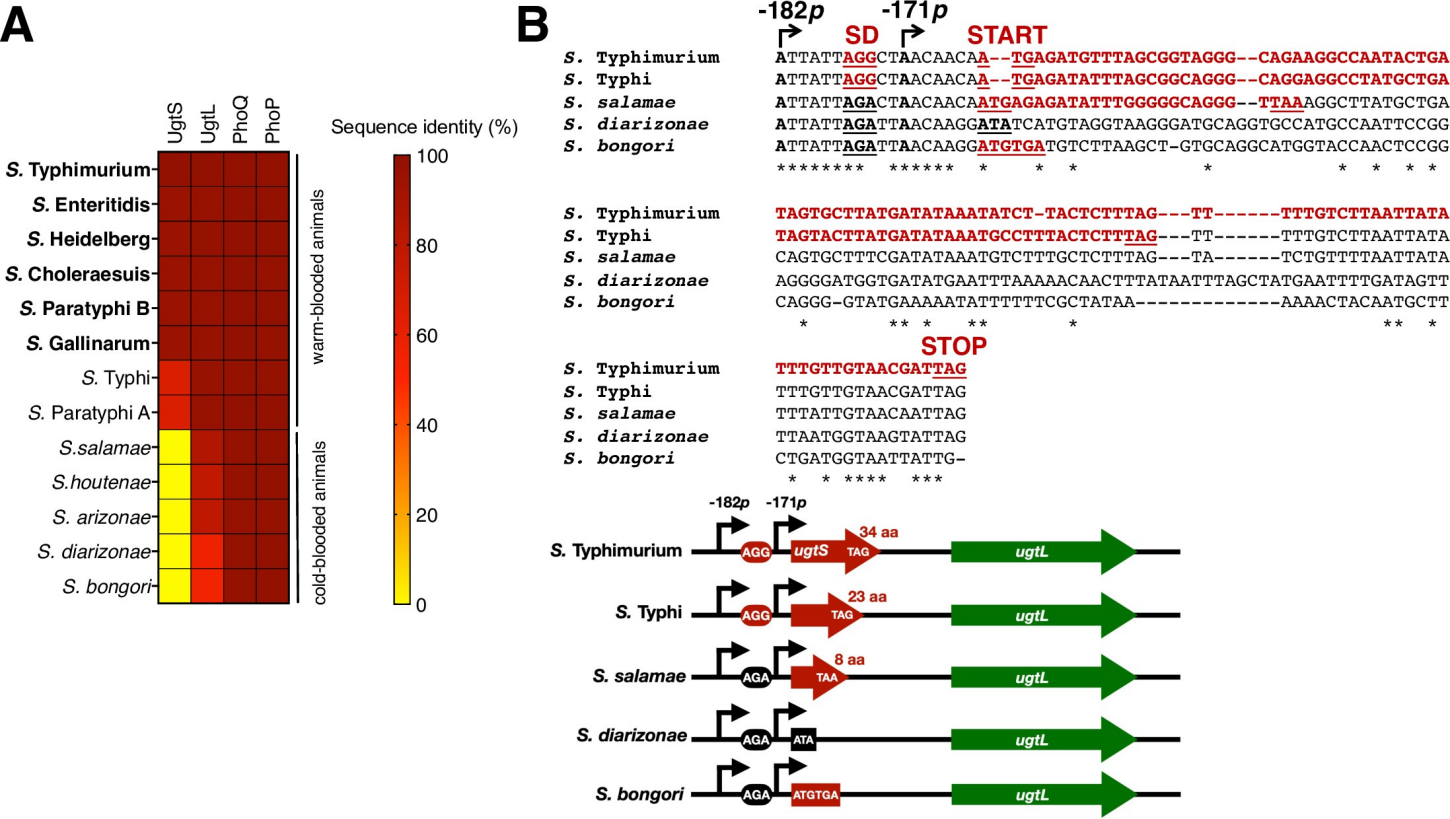
The *ugtS* gene is narrowly distributed in non-typhoidal *Salmonella* serovars infecting warm-blooded animals. (A) Heat map of UgtS, UgtL, PhoQ, and PhoP sequence identity among *Salmonella* species and serovars. Deduced amino acids sequences for each protein from *S*. *bongori* (NCTC12419), *S*. *enterica* subsp. *diarizonae* (SA20044251; *S*. *diarizonae*), *S*. *enterica* subsp. *arizonae* (RKS2980; *S*. *arizonae*), *S*. *enterica* subsp. *houtenae* (NCTC 7318; *S*. *houtenae*), *S*. *enterica* subsp. *salamae* (Locarno; *S*. *salamae)*, *S*. *enterica* subsp. *enterica* serovar Paratyphi A (ATCC11511; *S*. Paratyphi A), *S*. *enterica* subsp. *enterica* serovar Typhi (Ty2; *S*. Typhi), S. *enterica* subsp. *enterica* serovar Gallinarum (9184; *S*. Gallinarum), S. *enterica* subsp. *enterica* serovar Paratyphi B (SPB7; S. Paratyphi B), *S*. *enterica* subsp. *enterica* serovar Cholerasuis (SC-B67; *S*. Cholerasuis), *S*. *enterica* subsp. *enteric*a serovar Heidelberg (41578; *S*. Heidelberg), and *S*. *enterica* subsp. *enterica* serovar Enteritidis (P125109; *S*. Enteritidis) were compared to those of wild-type S. Typhimurium (14028s) using Protein BLAST. Non-typhoidal *Salmonella* serovars in which UgtS is conserved are indicated in bold. % identity values are displayed in color map. (B, *Top*) Alignment of the 5’ leader and coding region sequences of *ugtS* in the indicated *Salmonella* species and serovar. The -182*p* and -171*p* transcription start sites (TSSs) are indicated by arrows. The coding region and Shine-Dalgarno (SD) sequence (AGG) of *ugtS* are indicated in bold red. Degenerated Shine-Dalgarno sequences (AGA) and start codon (ATA) are indicated in bold black. The start (START) and stop (STOP) codons and Shine-Dalgarno sequences of *ugtS* are underlined. (B, *Bottom*) Schematic of the *ugtSugtL* gene cluster in the species aligned in (B, *Top*). aa, amino acids.

The UgtL protein is highly conserved (≥98% deduced shared amino acid sequence identity) in *S*. *enterica* serovars that, like serovar Typhimurium, infect a variety of warm-blooded animals and in which PhoP is required for virulence (Fig 7A). For instance, *phoP* inactivation in *S*. Gallinarum, *S*. Choleraesuis, and *S*. Typhi attenuates virulence in chickens, pigs, and humans, respectively [32–34].

We determined that the UgtS protein is highly conserved (≥97% shared identity in deduced amino acid sequences) among non-typhoidal *S*. *enterica* serovars that infect warm-blooded animals. In typhoidal serovars, the shared identity is reduced to 68% (Fig 7A) and the length of UgtS reduced from 34 to 23 amino acids (Fig 7B). The latter UgtS variant does not appear to be functional in reducing the PhoP-P-to-PhoP ratio because heterologous expression of the *S*. Typhi *ugtS* gene in the *ugtL-FLAG ugtSmutAUG S*. Typhimurium mutant failed to decrease the PhoP-P-to-PhoP ratio and UgtL protein amounts in mildly acidic pH (S2 Fig), behaving like the vector control (S2 Fig) and unlike the plasmid expressing the *S*. Typhimurium *ugtS* gene (S2 Fig), used as positive control. Of course, these results do not rule out the possibility of the shorter UgtS present in *S*. Typhi being functional in *S*. Typhi or having a function other than that established here for the *S*. Typhimurium UgtS: decreasing the PhoP-P-to-PhoP ratio in a UgtL-dependent manner.

UgtS is absent from the non-pathogenic *Salmonella* species *S*. *bongori* and from the *S*. *enterica* subspecies *salamae*, *arizonae*, *houtenae*, and *diarizonae*, all predominantly associated with cold-blooded animals [35,36] (Fig 7A). That is, remnants of the *ugtS* coding region lack sequences resembling a ribosome binding site (presence of a G to A single nucleotide polymorphism that also corresponds to the engineered substitution in the *ugtSmutSD-182*::*gfp* derivative; Fig 2A and 2D) and/or a start codon and/or have a premature stop codon (Fig 7B). In sum, the narrow distribution of the *ugtS* gene suggests that modulating the kinetics of the PhoP virulence program induction during infection via antagonization of the UgtL protein by UgtS benefits *S*. *enterica* serovars occupying specific habitats. The apparent absence of a donor outgroup for the *ugtS* gene in the examined *Salmonella* genomes (Fig 7A and 7B) also leaves open the possibility of *ugtS* having been created *de novo* rather than acquired horizontally, like the coexpressed *ugtL* gene [24].

## Discussion

Bacterial operons are generally transcribed as polycistronic mRNAs that enable the coordinated translation of the specified proteins, which most often participate in the same biological pathway [1–5]. We have now established that: (i) the *Salmonella*-specific PhoP-activated virulence gene *ugtL* is part of an operon with the upstream *ugtS* gene (Fig 1A); (ii) the *ugtSugtL* operon is transcribed as two distinct mRNAs (Figs 1A and 3A) that differ in the translatability of the upstream *ugtS* (Fig 2C); (iii) the two mRNAs are transcribed with different kinetics when *Salmonella* is inside macrophages (Fig 6C); (iv) the UgtS protein antagonizes UgtL’s ability to promote the phosphorylated (active) state of the master virulence regulator PhoP (Fig 4A, 4B and 4C); (v) the identified regulation controls the timing of a critical virulence program (Fig 6D); and (vi) the identified regulation is limited to a subset of *Salmonella* species and serovars that infect warm-blooded hosts (Fig 7A).

### Differential expression of genes within an operon

Typically, proteins specified in an operon are coordinately produced whereby overlap of the stop codon of an upstream open reading frame (ORF) with the start codon of the following ORF enables translational coupling of the two ORFs [37,38]. By contrast, the *ugtSugtL* bicistron is transcribed as two distinct mRNAs with 5’ leader regions of 171 and 182 nt in length. The differential expression of the two mRNAs enables independent production of the UgtS and UgtL proteins because only the longer mRNA includes the ribosome binding site for *ugt*S (Fig 3C). This mechanism differs from those mediated by small regulatory RNAs that selectively target specific genes within an operon for translational regulation or by altering the stability of selected portions of a polycistronic mRNA [39–43], and those resulting from differences in codon bias of individual genes [44,45].

### A *Salmonella-*specific small protein controls activation of the master virulence regulator

Proteins of 50 or fewer amino acids are increasingly recognized as regulators of key cellular processes [46,47], including signal transduction [29], nutrient and ion transport [21,22,48–51], and stress response [52]. They exert their regulatory functions through direct interaction with target proteins by using various mechanism of action, including regulated proteolysis [21,22,49], modulation of sensor kinase activity [29], regulation of the specificity, activity, and amounts of transporters [48,51,53,54], and stabilization of enzyme complexes [55].

We have now established the function of the novel small protein UgtS: controlling activation of the master virulence regulatory system PhoP/PhoQ [6,7,9,56]. UgtS binds to both the sensor PhoQ and its direct activator UgtL, a PhoQ-binding protein that promotes PhoQ autophosphorylation from ATP, thereby increasing the abundance of PhoP-P [15]. Because it is part of the same transcriptional units as *ugtL*, *ugtS* is also transcriptionally activated by PhoP. Thus, PhoP-P exerts feedback on itself by transcribing the *ugtSugtL* operon, the UgtS protein promoting negative feedback by reducing PhoP activity, and the UgtL protein exerting positive feedback by enhancing PhoQ autokinase activity, and thus PhoP activity. The differential production of the two *ugtSugtL* transcripts over time changes UgtS amounts, which dictates the kinetics with which PhoP-activated genes are transcribed when *S*. Typhimurium is inside macrophages (Fig 6D).

The activity of the *Salmonella-*specific UgtS protein is reminiscent, in part, of that exhibited by the PhoP-activated MgrB, a small protein that limits PhoP activation by inhibiting PhoQ’s autokinase activity [29,30]. However, UgtS and MgrB operate via distinct mechanisms: MgrB directly inhibits PhoQ autokinase activity [29], whereas UgtS antagonizes the PhoQ autophosphorylation enhancer UgtL [15] (Fig 4B), interacting with both the UgtL and PhoQ proteins (Fig 5). In addition, *ugtS* is *Salmonella*-specific, whereas *mgrB* is broadly distributed within the *Enterobacteriales*. Like the *Salmonella*-specific UgtL [15], the *E*. *coli*-specific SafA promotes PhoQ autophosphorylation [57,58], raising the possibility of *E*. coli specifying a SafA inhibitor analogous to UgtS in *S*. Typhimurium.

PhoP is necessary for transcription of both the *ugtSugtL*_*-182*_ and *ugtSugtL*_*-171*_ transcripts (Fig 3C). Accumulation of the longer transcript at early times inside macrophages (Fig 6C) allows *S*. Typhimurium to delay full PhoP activation because this mRNA enables *ugtS* translation (Fig 2C). Notably, wild-type and *ugtSmutAUG* mutant *S*. Typhimurium exhibited similar abundance of PhoP-activated transcripts at 1 h post internalization by macrophages (Fig 6D), reflecting that PhoP activation at this time is largely UgtL-independent [31]. By contrast, the *ugtSmutAUG* mutant had more PhoP-activated mRNAs than the wild-type at 6 h post internalization (Fig 6D), when the *ugtSugtL*_*-171*_ mRNA allows translation of *ugtL* but not *ugtS*, thus enabling PhoP activation [31] and susceptibility to UgtS action. These results indicate that the time at which an mRNA is made does not necessarily reflect the time at which a phenotype is observed. In agreement with this notion, activation of the PmrA/PmrB two-component system results in expression of PmrA-activated genes specifying proteins that modify the lipopolysaccharide (LPS), but the LPS modifications occur much later than the time of transcription of the genes specifying the LPS-modifying enzymes [59]. In contrast to what is observed inside macrophages (Fig 6C and 6D), the *ugtSugtL*_*-171*_ transcript accumulates at early time points (2–4 h) when *S*. Typhimurium is grown in defined laboratory media of mildly acidic pH (Fig 3A), a condition that produces sufficient UgtL to render PhoP activation susceptible to UgtS action (Figs 4A and 6A).

### Reciprocal regulation between ancestral and horizontally acquired genes

Horizontally acquired genes and their products are typically subjected to more regulation than ancestral genes, a feature that has been ascribed to the need to ensure their proper integration into the existing regulatory networks of an ancestral genome [60–65]. This is reflected by the extensive and multilayered regulation of the horizontally acquired *ugtSugtL* bicistron and of the proteins it specifies. That is, *ugtL* transcription requires the SlyA and SsrB proteins to relieve *ugtL* silencing by H-NS [31,66] and PhoP to recruit RNA polymerase [14,31]; and *ugtL* translation requires the RNA chaperone CspC to disrupt a secondary structure that sequesters *ugtL*’s Shine-Dalgarno sequence [67].

We have now uncovered two additional layers of *ugtSugtL* regulation: first, the small protein UgtS antagonizes UgtL’s ability to activate PhoP (Figs 4 and 6A, 6B and 6D), and second, *ugtSugtL* is transcribed as two different mRNAs that differ in their ability to allow *ugtS* translation (Fig 2). Intriguingly, *ugtSugtL*_*-182*_ and *ugtSugtL*_*-171*_ mRNAs are both produced in a PhoP-dependent manner (Fig 3D) but display different accumulation inside macrophages (Fig 6C). Whereas the *ugtSugtL*_*-17*1_ transcript peaks at later times, like other PhoP-activated genes [31], *ugtSugtL*_*-182*_ levels remain steady over time (Fig 6C), suggesting that a regulator(s) other than PhoP responding to a macrophage signal(s) other than acidic pH regulates its expression.

Our findings unveiled a reciprocal regulation between ancestral and horizontally acquired factors. That is, the ancestral regulator PhoP directly promotes transcription of the horizontally acquired *ugtSugtL* operon, thereby governing synthesis of the UgtS and UgtL proteins which, in turn, modulate PhoP activity. One horizontally acquired protein, UgtL, enhances the activity of the ancestral regulator PhoP, while another horizontally acquired small protein, UgtS, hampers it. These reciprocal regulations (between ancestral and horizontally acquired factors) enable *S*. Typhimurium to time its virulence program during infection.

(Please note that the apparent absence of donor outgroup for the *ugtS* gene in the examined *Salmonella* genomes (Fig 7A and 7B) also leaves open the possibility of *ugtS* having been created *de novo* rather than acquired horizontally, like the co-expressed *ugtL* gene [24].)

### Concluding remarks

Our findings demonstrate that bacterial pathogenesis entails foreign genes controlling ancestral regulators to control the expression of virulence determinants in novel environments and for specific time spans.

## Materials and methods

### Bacterial strains, plasmids, primers, and growth conditions

Bacterial strains and plasmids used in this study are listed in S1 Table; oligonucleotide sequences are presented in S2 Table. Single gene knockouts and deletions were carried out as described [68]. Mutations generated by this approach were subsequently moved into clean genetic backgrounds via phage P22-mediated transduction as described [69].

Bacteria were grown at 37°C in Luria-Bertani broth (LB) or N-minimal medium pH 4.9 [70] supplemented with 0.1% casamino acids, 38 mM glycerol, and the indicated concentrations of MgCl_2_. *E*. *coli* DH5α was used as the host for the preparation of plasmid DNA. Ampicillin was used at 50 μg/mL, kanamycin at 50 μg/mL, chloramphenicol at 20 μg/mL, and tetracycline at 10 μg/mL.

### Strain construction

Mutant strains were constructed using the one-step inactivation method [68] with pKD3 or pKD4 plasmid DNA as template. When required, plasmid pCP20 [68] was used to remove antibiotic-resistance markers flanked by FRT sites.

To generate the *ugtSmutAUG* strain (HS1119), a PCR product was generated with primers W3781 and W3782 using the pSLC-242 plasmid [71] as template. The resulting PCR product was then integrated into the chromosome of wild-type *S*. *enterica* (14028s) via the one-step inactivation method [68] using the pKD46 plasmid. Recombinant bacteria containing the insertion were selected on LB supplemented with 20 μg/ml chloramphenicol at 30°C. This insertion was subsequently replaced via a second pKD46-mediated recombination of pre-annealed W3783 and W3784 primers into the chromosome. Bacteria were incubated for 3 h as described [71] and plated on N-minimal medium agar plates [72] containing 50 μM glutamate, 50 μM histidine, 50 μM leucine, 100 μM methionine, 100 μM glutamine, 10 mM MgCl_2_, and 30 mM rhamnose as the sole carbon source. The allele replacement was confirmed by DNA sequencing of a PCR product generated with primers W3560 and W43621.

To construct the *ugtL*::*Cm*^*R*^ *ugtSmutAUG* strain (HS1548), a PCR product generated with primers W4095-W4463 using the pKD3 plasmid as a template was integrated into the *ugtSmutAUG* strain via the one-step inactivation method [68] using plasmid pKD46. The *ugtL*::*Cm*^*R*^ *ugtSmutAUG* allele was subsequently moved into wild-type *S*. Typhimurium *(*strain 14028s) via phage P22-mediated transduction as described [69].

To construct the *ugtS-SPA*::*Km*^*R*^ strain (HS1170), a PCR product generated with primers W3999-W4000 using plasmid pJL148 as a template was integrated into wild-type *S*. Typhimurium (strain 14028s) via the one-step inactivation method [68] using plasmid pKD46. Cassette insertion was confirmed by PCR with primers W4095 and W4096. The *ugtS-SPA*::*Km*^*R*^ allele was subsequently moved into wild-type *S*. Typhimurium (strain 14028s) via phage P22-mediated transduction as described [69].

To construct the *ugtS-SPA*::*Km*^*R*^ *phoP*::Tn*10* strain (HS1178), the *phoP*::Tn*10* allele was moved from strain MS7953s (*phoP*::Tn*10*) to strain HS1170 (*att*Tn*7*::*ugtS-SPA*) via phage P22-mediated transduction as described [69].

To construct the *ugtS-SPA* strain (HS1536), a PCR product generated with primers W4466 and W4000 using plasmid pSLC-242 [71] as a template was integrated into the chromosome of the *ugtS-SPA*::*Km*^*R*^ strain (HS1170) via the one-step inactivation method [68] using plasmid pKD46. Recombinant bacteria containing the insertion were selected on LB supplemented with 20 μg/ml chloramphenicol at 30°C. This insertion was subsequently replaced via a second pKD46-mediated recombination of pre-annealed W4467 and W4468 primers into the chromosome. Bacteria were incubated for 3 h as described [71] and plated on N-minimal medium agar plates [72] containing 50 μM glutamate, 50 μM histidine, 50 μM leucine, 100 μM methionine, 100 μM glutamine, 10 mM MgCl_2_, and 30 mM rhamnose as the sole carbon source. The allele replacement was confirmed by DNA sequencing of a PCR product generated with primers W4001 and W4002.

To generate the *att*Tn7-*ugtS-SPA* strain (HS1795), pGRG25-*ugtS-SPA* (see construction details in the section below) was transformed into wild-type *S*. Typhimurium (strain 14028s), and integration into *att*Tn*7* site was performed as described [73] with some modifications. The pGRG25-*ugtS-SPA*-carrying strain was incubated overnight at 30°C in LB supplemented with 0.05% arabinose. The insertion was confirmed by DNA sequencing of a PCR product generated with primers W4636 and W4637.

To construct the *plac1-6_-12ugtL*::*Cm*^*R*^ strain (JC1358), a PCR product generated with primers 16655–16658 using the pKD3 plasmid as a template was integrated into wild-type *S*. Typhimurium (strain 14028s) via the one-step inactivation method [68] using plasmid pKD46. The insertion was confirmed by PCR with primers W3558 and W3559.

To construct the *plac1-6_-12ugtL*::FRT strain (JC1360), the pCP20 helper plasmid [68] was used to remove the Cm^R^ marker from strain JC1358 (*Plac1-6_-12ugtL*::*Cm*^*R*^).

To construct the *plac1-6_-12ugtL-FLAG*::*Km*^*R*^ strain (JC1362), a PCR product generated with primers 16686 and 16687 using the pKD4 plasmid as a template was integrated into the chromosome of strain JC1360 (*Plac1-6_-12ugtL*::FRT) via the one-step inactivation method [68] using plasmid pKD46. The insertion was confirmed by PCR with primers W3558 and W3559.

To construct the *plac1-6_-12ugtL*_*1-89truncation*_-FLAG::*Km*^*R*^ strain (JC1414), a PCR product generated with primers 16864 and 16687 using plasmid pKD4 as template was integrated into the chromosome of strain *plac1-6_-12ugtL*::FRT strain (JC1360) via the one-step inactivation method [68] using plasmid pKD46. The insertion was confirmed by PCR with primers W3558 and W3559.

To construct the *ugtL*-*FLAG*::*Cm*^*R*^ strain (HS1189), a PCR product generated with primers W3871 and W3872 using plasmid pKD3 as a template was integrated into wild-type *S*. Typhimurium (strain 14028s) via the one-step inactivation method [68] using plasmid pKD46. The insertion was confirmed by PCR with primers W3873 and W3874. The *ugtL*-*FLAG*::*Cm*^*R*^ allele was subsequently moved into wild-type *S*. Typhimurium (strain 14028s) via phage P22-mediated transduction as described [69].

To construct the *att*Tn*7*::*ugtS-SPA ugtL*-*FLAG*::*Cm*^*R*^ strain (HS1940), the *ugtL*-*FLAG*::*Cm*^*R*^ allele was moved from strain HS1189 (*ugtL*-*FLAG*::*Cm*^*R*^) to strain HS1795 (*att*Tn*7*::*ugtS-SPA*) via phage P22-mediated transduction as described [69].

To construct the *att*Tn*7*::*ugtS-SPA phoP*::Tn*10* strain (HS1823), the *phoP*::Tn*10* allele was moved from strain MS7953s (*phoP*::Tn*10*) to strain HS1795 (*att*Tn*7*::*ugtS-SPA*) via phage P22-mediated transduction as described [69].

To construct the *ugtL*-*FLAG*::*Cm*^*R*^ *ugtSmutAUG* strain (HS1198), a PCR product generated with primers W3871 and W3872 using plasmid pKD3 as template was integrated into strain HS1119 (*ugtSmutAUG*) via the one-step inactivation method [68] using plasmid pKD46. The insertion was confirmed by PCR with primers W3873 and W3874. The *ugtL*-*FLAG*::*Cm*^*R*^ *ugtSmutAUG* allele was subsequently moved into wild-type *S*. Typhimurium (strain 14028s) via phage P22-mediated transduction as described [69].

To generate the *ugtL*-*FLAG*::FRT *ugtSmutAUG* strain (HS1207), the pCP20 helper plasmid [68] was used to remove the *Cm*^*R*^ marker from strain HS1198 (*ugtL*-*FLAG*::*Cm*^*R*^ *ugtSmutAUG*).

To generate the *ugtL*-*FLAG*::FRT *ugtSmutAUG mgrB*::*Cm*^*R*^ strain (HS2414), the *mgrB*::*Cm*^*R*^ allele was moved from strain JC969 (*hns*-*FLAG*::FRT *mgrB*::*Cm*^*R*^) to strain HS1207 (*ugtL*-*FLAG*::FRT *ugtSmutAUG*) via phage P22-mediated transduction as described [69].

### Construction of plasmids

To construct pUHE-UgtS, primers W3859 and W3860 were used to amplify *ugtS* -18 to +120 region (relative to *ugtS* ATG start codon) (*ugtL* -182 to -45 region relative to *ugtL* ATG start codon) using wild-type *S*. Typhimurium (strain 14028s) genomic DNA as template. The resulting PCR product was digested with EcoRI and HindIII and ligated into pUHE-21 plasmid [74] digested with the same restriction enzymes. The ligation reaction was transformed into DH5α cells by electroporation. The identity of the *ugtS* insert was verified by DNA sequencing using primers W912-W913.

To construct pUHE-UgtS_Typhi_, primers 17859–17860 were used to amplify *ugtS* -18 to +87 region (relative to *ugtS* ATG start codon) (*ugtL* -183 to -79 region relative to *ugtL* ATG start codon) using *Salmonella enterica* serovar Typhi strain ISP2825 genomic DNA as template. The resulting PCR product was digested with EcoRI and HindIII and ligated into pUHE-21 plasmid [74] digested with the same restriction enzymes. The ligation reaction was transformed into DH5α cells by electroporation. The identity of *ugtS*_*Typhi*_ insert was verified by DNA sequencing using primers W912-W913.

To construct pXG10sf-*ugtS-182*, primers W3503-W3786 were used to amplify *ugtS* -18 to +102 region (relative to *ugtS* ATG start codon) (*ugtL* -182 to -63 region relative to *ugtL* ATG start codon) using wild-type *S*. Typhimurium (strain 14028s) genomic DNA as template. The resulting product was then digested with NheI and NsiI and ligated into pXG10sf digested with the same enzymes. The ligation reaction was transformed into DH5α cells by electroporation. The identity of the *ugtS* insert was verified by DNA sequencing using primers W1332-W1333.

To construct pXG10sf-*ugtS-171*, primers W3364-W3786 were used to amplify *ugtS* -7 to +102 region (relative to *ugtS* ATG start codon) (*ugtL* -171 to -63 region relative to *ugtL* ATG start codon) using wild-type *S*. Typhimurium (strain 14028s) genomic DNA as template. The resulting product was then digested with NheI and NsiI and ligated into pXG10sf digested with the same enzymes. The ligation reaction was transformed into DH5α cells by electroporation. The identity of the *ugtS* insert was verified by DNA sequencing using primers W1332-W1333.

To construct pXG10sf-*ugtSmutAUG-182*, primers W3785 and W3786 were used to amplify *ugtSmutAUG* -18 to +102 region (relative to *ugtS* ATG start codon) (*ugtL* -182 to -63 region relative to *ugtL* ATG start codon) using wild-type *S*. Typhimurium (strain 14028s) genomic DNA as template. The resulting product was then digested with NheI and NsiI and ligated into pXG10sf digested with the same enzymes. The ligation reaction was transformed into DH5α cells by electroporation. The identity of the *ugtS* insert was verified by DNA sequencing using primers W1332-W1333.

To construct pXG10sf-*ugtSmutSD-182*, primers 17857-W3786 were used to amplify *ugtSmutSD* -18 to +102 region (relative to *ugtS* ATG start codon) (*ugtL* -182 to -63 region relative to *ugtL* ATG start codon) using wild-type *S*. Typhimurium (strain 14028s) genomic DNA as template. The resulting product was then digested with NheI and NsiI and ligated into pXG10sf digested with the same enzymes. The ligation reaction was transformed into DH5α cells by electroporation. The identity of the *ugtS* insert was verified by DNA sequencing using primers W1332 and W1333.

To construct pGRG25-*ugtS-SPA*, primers W4634 and W4635 were used to amplify *ugtS*-*SPA* using a cell lysate of HS1536 strain (*ugtS-SPA*) as a template. The resulting product was then digested with XhoI and ligated into pGRG25 digested with the same enzymes. The ligation reaction was transformed into DH5α cells by electroporation. The identity of the *ugtS-SPA* insert was verified by DNA sequencing using primers W4638 and W4639.

### Pull-down assays with proteins synthesized using an *in vitro* transcription-translation system

Pull-down assays were performed as described [15] with some modifications. Proteins were produced from DNA templates by *in vitro* synthesis using the PURExpress system (New England Biolabs). To synthesize the DNA templates, primer pairs W4220 and W4804 (*ugtS-HA*), W4222 and W4302 (*ugtL-FLAG*), W4319 and W4320 (*phoQ-FLAG*), and W4932 and W4933 (*dppC-FLAG)* were used. Synthesized proteins were mixed in 500 μl of tris-buffered saline (TBS) containing proteoliposomes (0.12 mg/ml) and incubated at room temperature for 2 h. Samples were then pulled down with anti-HA magnetic beads (Thermo Scientific) at room temperature for 2 h. Samples were then analyzed by Western blot with antibodies directed to the FLAG (Abcam) or HA (Sigma) epitopes. Where indicated, images were quantified using ImageLab software (Biorad).

### Western blot assay

Overnight cultures of cells grown in N-minimal medium (pH 7.7) [70] supplemented with 10 mM MgCl_2_ were diluted 1/50 in mildly acidic pH N-minimal medium (pH 4.9, 1 mM MgCl_2_), and cells were grown for the indicated times. Media were supplemented with 20 μg/ml chloramphenicol for experiments with strains carrying pXG10sf constructs. To extract total proteins, cells were precipitated with trichloroacetic acid (5% total volume) and washed with 80% acetone. Samples were resuspended in NuPAGE LDS sample buffer (ThermoFisher Scientific) and normalized according to the OD_600_. Protein samples intended to be run on a Phos-tag gel were extracted with formic acid as previously described [75]. Protein samples were run on NuPAGE 4–12% bis-tris protein gels (ThermoFisher Scientific) and transferred to nitrocellulose membrane using iBlot Gel Transfer Device (ThermoFisher Scientific). Membranes were blocked with 5% milk solution in TBST for 1 h. Membranes were probed with 1:5000 dilution of mouse anti-GFP (Sigma), mouse anti-FLAG (Sigma), rabbit anti-FLAG (ThermoFisher Scientific; in Fig 4C), rabbit anti-HA (Sigma) or mouse anti-RpoB (BioLegend). Secondary horseradish peroxidase-conjugated anti-rabbit (GE healthcare) or anti-mouse (Promega) was used at 1:5000 dilution. The blots were developed with the Amersham ECL Western blotting detection reagents (GE Healthcare) or SuperSignal West Femto chemiluminescent system (Pierce). Images were acquired with LAS-4000 imager (GE Healthcare). When required, images were quantified using ImageLab software (Biorad).

### *In vivo* detection of phosphorylated PhoP

Overnight cultures of cells grown in N-minimal medium (pH 7.7) [70] supplemented with 10 mM MgCl_2_ were diluted 1/50 in mildly acidic pH N-minimal medium (pH 4.9, 1 mM MgCl_2_), and cells were grown for the indicated times. Whole-cell extracts were prepared as described [75]. Samples were run on 12.5% polyacrylamide gels containing acrylamide–Phos-tag ligand (Wako Laboratory Chemicals) in standard running buffer [0.4% (w/v) SDS, 25 mM tris, 192 mM glycine] at 150 V at 4°C for 4 h, transferred to nitrocellulose membranes, and analyzed by immunoblotting using polyclonal rabbit antibodies recognizing PhoP (1,4000) and polyclonal mouse antibodies recognizing AtpB (Abcam) (1,5000). The blots were developed with the SuperSignal West Femto chemiluminescent reagents (Pierce). Images were acquired with LAS-4000 imager (GE Healthcare). Where indicated, images were quantified using ImageLab software (Biorad).

### Primer extension analysis

Overnight cultures of cells grown in N-minimal medium (pH 7.7) [70] supplemented with 10 mM MgCl_2_ were diluted 1/50 in mildly acidic pH N-minimal medium (pH 4.9, 1 mM MgCl_2_), and cells were grown for the indicated times. Total RNA was extracted using the hot phenol procedure as previously described [76]. Total RNA from bacterial cells inside macrophages was isolated using Trizol according to the manufacturer’s directions (ThermoFisher Scientific). Primer extension reactions were then performed as previously described [77] using 10–20 μg of total RNA and either the primer W4469 annealing with *ugtL* -105 to -86 region (relative to *ugtL* ATG start codon) or the primer W4055 annealing with *ugtL* -122 to -141 region (relative to *ugtL* ATG start codon). Primer extension reactions were run together with a template-specific sequencing ladder generated with either W4669 or W3560 primers and a DNA template corresponding to *ugtL* -297 to +41 region (relative to *ugtL* ATG start codon) amplified with primers W3559-W3560 using wild-type *S*. Typhimurium (strain 14028s) genomic DNA as template. Where indicated, images were quantified using ImageLab software (Biorad).

### Total RNA extraction from bacterial cells inside macrophages

The murine-derived macrophage-like cell line J774A.1 was cultured in Dulbecco’s modified Eagle’s medium (DMEM; Life Technologies) supplemented with 10% FBS (Life Technologies) at 37°C under 5% CO_2_. Confluent monolayers for infection with bacteria were prepared in 6-well tissue culture plates. Each well was seeded with 10^6^ cells suspended in DMEM/10% FBS and incubated at 37°C under 5% CO_2_. Bacterial cells grown overnight in LB broth were washed two times with DMEM-10% FBS, suspended in pre-warmed DMEM-10% FBS, and then added to the cell monolayer at a multiplicity of infection (MOI) of 10. To promote bacterial internalization, plates were centrifuged at 1000 *g* for 3 min. Plates were then incubated 30 min at 37°C (defined as time 0 h). Cells were then washed three times with DPBS and extracellular bacteria were killed with 100 μg/ml gentamicin. The incubation was pursued for 1 h (defined as time 1 h), cells washed with DPBS, and the medium replaced with medium containing 10 μg/ml gentamicin. Incubation was then continued for a further 5 h (defined as time 6 h). For each time point (0, 1 and 6 h), cells were washed with DPBS and total RNA from inside macrophages isolated using Trizol according to the manufacturer’s directions (ThermoFisher Scientific).

### Quantitative RT-PCR

Overnight cultures were grown in N-minimal medium (pH 7.7) [70] supplemented with 10 mM MgCl_2_ were diluted 1/50 in mildly acidic pH N-minimal medium (pH 4.9, 1 mM MgCl_2_), and cells were grown for the indicated times. Total RNA was extracted using the hot phenol procedure as previously described [76]. Total RNA from bacterial cells inside macrophages was isolated using Trizol according to the manufacturer’s directions (ThermoFisher Scientific). Quantification of transcripts was carried out by qRT–PCR using SYBR Green PCR Master Mix (Applied Biosystems) in QuantStudio 6 Flex real-time PCR system (Applied Biosystems). The relative amount of mRNA was determined using a standard curve obtained by PCR with serially diluted genomic DNA from wild-type *S*. Typhimurium (strain 14028s), and results were normalized to the amounts of the *rrs* gene. The mRNA amounts of the *rrs*, *mgtC*, *pagC*, *pcgL*, *pmrD*, and *ugtL* genes were measured using the following primer pairs (*rrs*, W1883-W1884; *mgtC*, 6962–6963; *pagC*, 6964–6965; *pcgL*, 6627–6628; *pmrD*; 14514–14515; and *ugtL*, W856-W857). Data shown are an average from at least three independent experiments.

## Supporting information

S1 Fig UgtS reduces PhoP activity independently of the MgrB protein. Western blot analysis of extracts prepared from *ugtL-FLAG ugtSmutAUG* (HS1207) and *ugtL-FLAG ugtSmutAUG mgrB* (HS2414) *S*. Typhimurium harboring plasmid pUgtS or pVector (empty pUHE-21 vector) grown for 4 h (mid-log phase) in N-minimal acidic pH medium (pH 4.9, 1 mM MgCl_2_) supplemented with 0.2 mM IPTG before inoculation. Samples were analyzed using Phos-tag gels with antibodies directed to the PhoP and AtpB proteins (upper panel) and SDS-PAGE with antibodies directed to the FLAG epitope or RpoB protein (lower panel). Data are representative of two independent experiments, which gave similar results.
(TIF)

S2 Fig The 23-amino acid UgtS variant from *S*. Typhi fails to reduce PhoP activity in *S*. Typhimurium. (*Left*) Western blot analysis of extracts prepared from *ugtL-FLAG ugtSmutAUG* (HS1207) *S*. Typhimurium harboring pUgtS, pUgtS_Typhi_ (pUHE-21 expressing the UgtS variant from *S*. Typhi) or pVector (empty pUHE-21 vector) grown for 4 h (mid-log phase) in N-minimal acidic pH medium (pH 4.9, 1 mM MgCl_2_) supplemented with 0.5 mM IPTG before inoculation. Samples were analyzed using Phos-tag gels with antibodies directed to the PhoP and AtpB proteins (upper panel) and SDS-PAGE with antibodies directed to the FLAG epitope or RpoB protein (lower panel). Data are representative of two independent experiments, which gave similar results. (*Right*) UgtS amino acid sequence conservation in *S*. Typhi (*S*. *enterica* subsp. *enterica* serovar Typhi strain Ty2). The percentage of identity of *S*. Typhi’s UgtS with *S*. Typhimurium’s (14028s) is indicated. The predicted transmembrane domain (predicted by TMpred [78]) is highlighted in gray.
(TIF)

S3 Fig Primer extension analysis of *ugtSugtL*_*-182*_ and *ugtSugtL*_*-171*_ transcripts amounts from wild-type (14028s) and *ugtSmutAUG* (HS1119) *S*. Typhimurium inside J774A.1 macrophages at the indicated times (low and high exposures gel images). Gel images of Fig 6C with low and high exposures are provided to better discern the DNA sequencing ladder bands.
(TIF)

S1 Table Bacterial strains and plasmids used in this study. (DOCX)

S2 Table Oligonucleotides sequences used in this study. (DOCX)

S1 Data Excel spreadsheet of the numerical values for the bands quantification in Fig 2D. (XLSX)

S2 Data Excel spreadsheet of the numerical values for the bands quantification in Fig 3A. (XLSX)

S3 Data Excel spreadsheet of the numerical values for the bands quantification in Fig 3B. (XLSX)

S4 Data Excel spreadsheet of the numerical values for the bands quantification in Fig 3C. (XLSX)

S5 Data Excel spreadsheet of the numerical values for the bands quantification in Fig 3D. (XLSX)

S6 Data Excel spreadsheet of the numerical values for the bands quantification in Fig 3E. (XLSX)

S7 Data Excel spreadsheet of the numerical values for the bands quantification in Fig 4A. (XLSX)

S8 Data Excel spreadsheet of the numerical values for the bands quantification in Fig 4B. (XLSX)

S9 Data Excel spreadsheet of the numerical values for the bands quantification in Fig 4C. (XLSX)

S10 Data Excel spreadsheet of the numerical values for the bands quantification in Fig 6A. (XLSX)

S11 Data Prism spreadsheet of the numerical values underlying the data presented in Fig 6B. Statistical analysis details are also included.
(PZFX)

S12 Data Prism spreadsheet of the numerical values underlying the data presented in Fig 6D. Statistical analysis details are also included.
(PZFX)
