# Supplementary figures and images for: Differential synthesis of novel small protein times Salmonella virulence program
Source: PLoS Genet. 2022 Mar 4;18(3):e1010074. doi: 10.1371/journal.pgen.1010074 (PMC8896665; doi:10.1371/journal.pgen.1010074)

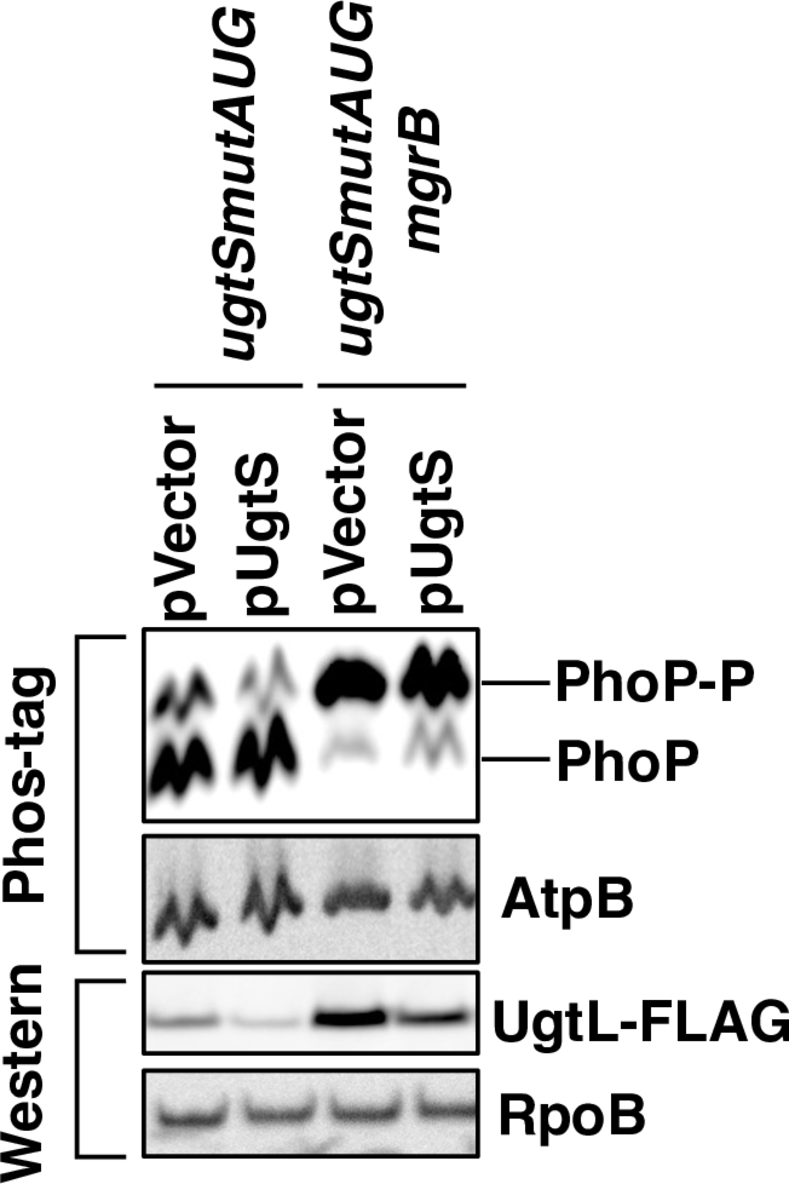

Supplement: S1 Fig — Western blot analysis of extracts prepared from ugtL-FLAG ugtSmutAUG (HS1207) and ugtL-FLAG ugtSmutAUG mgrB (HS2414) S. Typhimurium harboring plasmid pUgtS or pVector (empty pUHE-21 vector) grown for 4 h (mid-log phase) in N-minimal acidic pH medium (pH 4.9, 1 mM MgCl2) supplemented with 0.2 mM IPTG before inoculation. Samples were analyzed using Phos-tag gels with antibodies directed to the PhoP and AtpB proteins (upper panel) and SDS-PAGE with antibodies directed to the FLAG epitope or RpoB protein (lower panel). Data are representative of two independent experiments, which gave similar results. (TIF) [file pgen.1010074.s001.tif]

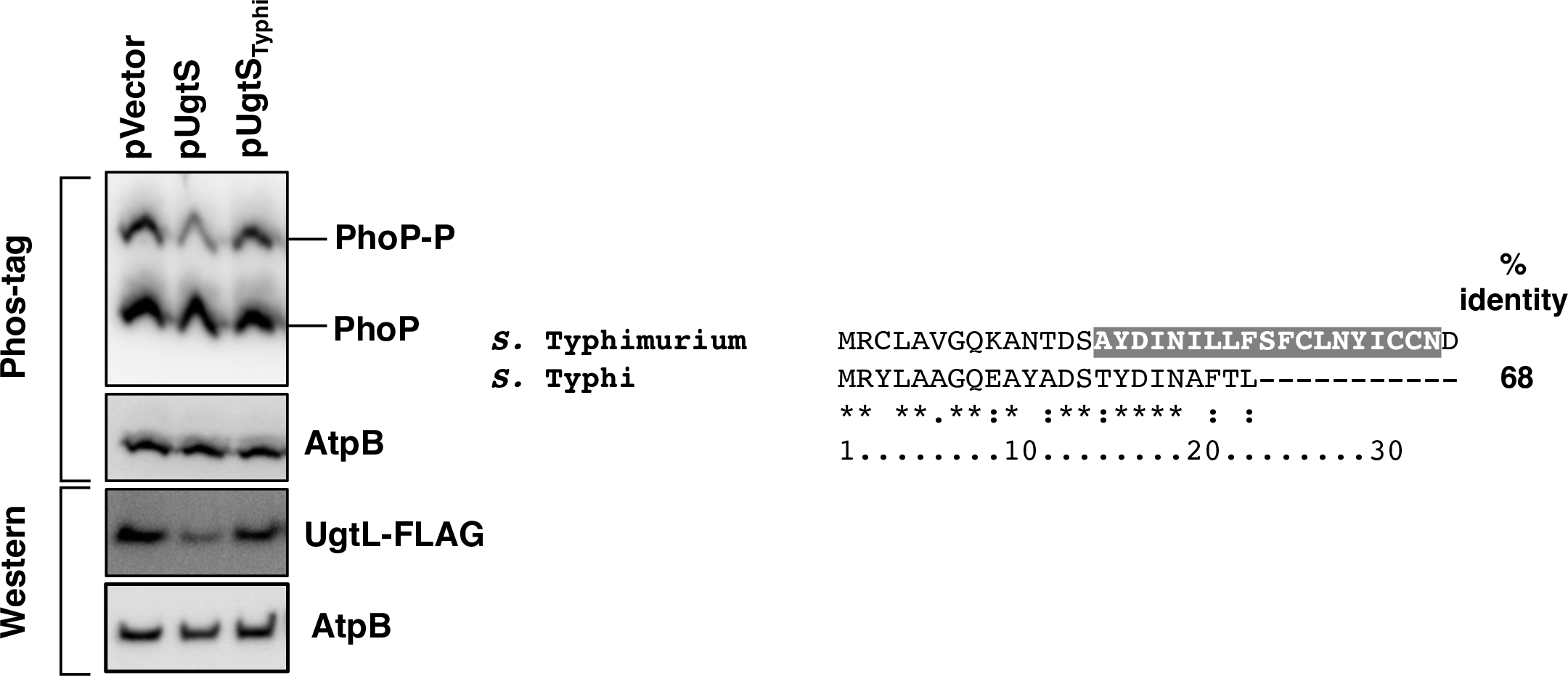

Supplement: S2 Fig — (Left) Western blot analysis of extracts prepared from ugtL-FLAG ugtSmutAUG (HS1207) S. Typhimurium harboring pUgtS, pUgtSTyphi (pUHE-21 expressing the UgtS variant from S. Typhi) or pVector (empty pUHE-21 vector) grown for 4 h (mid-log phase) in N-minimal acidic pH medium (pH 4.9, 1 mM MgCl2) supplemented with 0.5 mM IPTG before inoculation. Samples were analyzed using Phos-tag gels with antibodies directed to the PhoP and AtpB proteins (upper panel) and SDS-PAGE with antibodies directed to the FLAG epitope or RpoB protein (lower panel). Data are representative of two independent experiments, which gave similar results. (Right) UgtS amino acid sequence conservation in S. Typhi (S. enterica subsp. enterica serovar Typhi strain Ty2). The percentage of identity of S. Typhi’s UgtS with S. Typhimurium’s (14028s) is indicated. The predicted transmembrane domain (predicted by TMpred [78]) is highlighted in gray. (TIF) [file pgen.1010074.s002.tif]

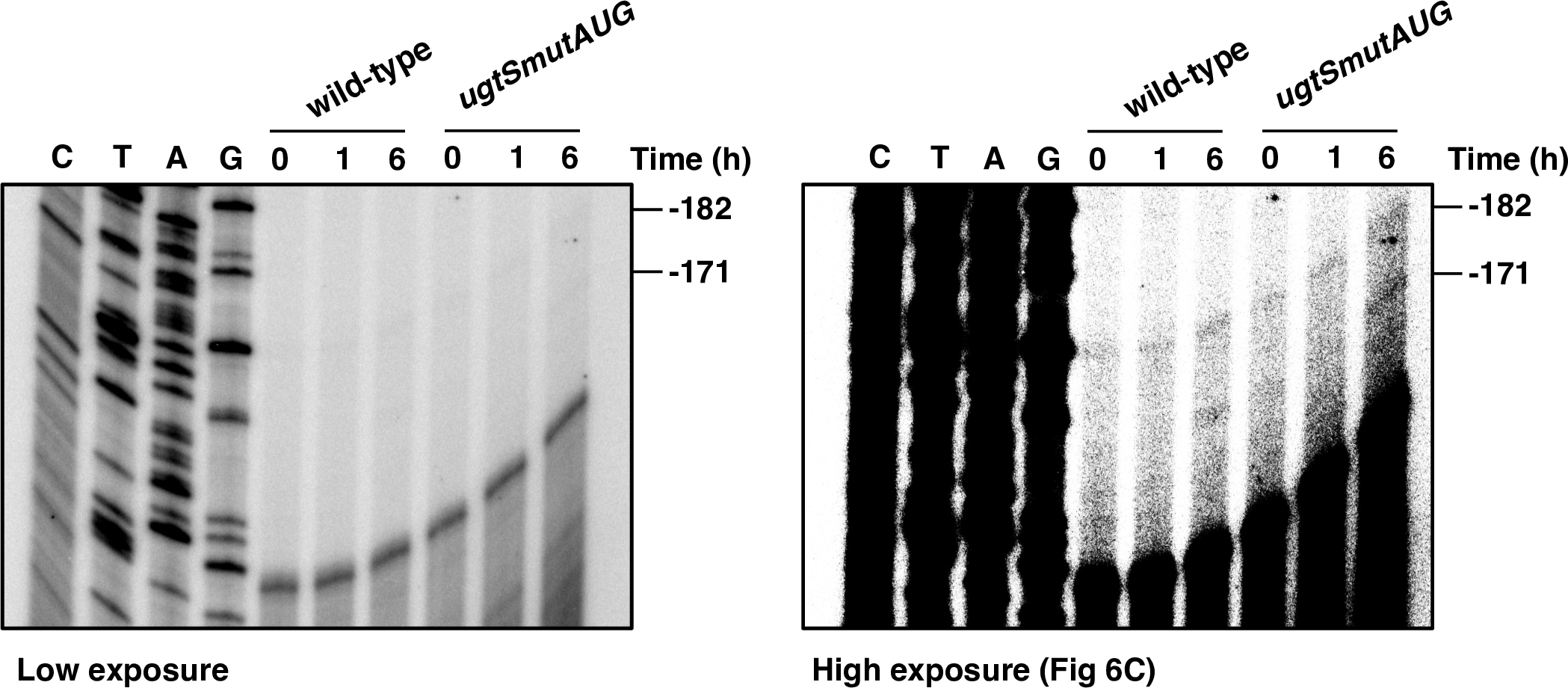

Supplement: S3 Fig — Gel images of Fig 6C with low and high exposures are provided to better discern the DNA sequencing ladder bands. (TIF) [file pgen.1010074.s003.tif]
