# Supplementary material for: Differential synthesis of novel small protein times Salmonella virulence program
Source: PLoS Genet. 2022 Mar 4;18(3):e1010074. doi: 10.1371/journal.pgen.1010074 (PMC8896665; doi:10.1371/journal.pgen.1010074)
Supplement: S1 Table — (DOCX) [file pgen.1010074.s004.docx]

**S1 Table.** Bacterial strains and plasmids used in this study

| **Strains** | **Relevant characteristics** | **Source** |
| --- | --- | --- |
| ***Escherichia coli*** |  |  |
| DH5α | Host strain used for generation and propagation of plasmid constructs | [1] |
| ***Salmonella enterica* serovar Typhimurium** |  |  |
| 14028s | Wild-type | [2] |
| MS7953s | *phoP*::Tn10 | [3] |
| HS1119 | *ugtSmutAUG* | This study |
| HS1170 | *ugtS-SPA*::*Km^R^* | This study |
| HS1178 | *ugtS-SPA*::Km^R^ *phoP*::Tn*10* | This study |
| HS1189 | *ugtL-FLAG*::*Cm^R^* | This study |
| HS1198 | *ugtL-FLAG*::*Cm^R^ ugtSmutAUG* | This study |
| HS1207 | *ugtL-FLAG*::FRT *ugtSmutAUG* | This study |
| HS1536 | *ugtS-SPA* | This study |
| HS1548 | Δ*ugtL*::Cm^R^ *ugtSmutAUG* | This study |
| HS1795 | *att*Tn*7*::*ugtS-SPA* | This study |
| HS1823 | *att*Tn*7*::*ugtS-SPA phoP*::Tn*10* | This study |
| HS1940 | *att*Tn*7*::*ugtS-SPA ugtL-FLAG*::*Cm^R^* | This study |
| HS2414 | *ugtL-FLAG*::FRT *ugtSmutAUG* Δ*mgrB*::*Cm^R^* | This study |
| JC969 | *hns-FLAG*::FRT Δ*mgrB*::*Cm^R^* | [4] |
| JC1358 | *Plac1-6_-12ugtL*::*Cm^R^* | This study |
| JC1360 | *Plac1-6_-12ugtL*::FRT | This study |
| JC1362 | P*lac1-6_-12ugtL-FLAG*::*Km^R^* | This study |
| JC1414 | P*lac1-6_-12ugtL_1-89_*-FLAG::*Km^R^* | This study |
| **Plasmids** | **Relevant characteristics** | **Source** |
| pCP20 | rep_pSC101_^ts^ λ cI857 FLP *Amp^R^ Cm^R^* | [5] |
| pGRG25 | rep_pSC101_^ts^ *Amp^R^* | [6] |
| pJL148 | *Km^R^* | [7] |
| pKD3 | rep_R6Kg_ *Amp^R^* FRT *Cm^R^* FRT | [5] |
| pKD4 | rep_R6Kg_ *Amp^R^* FRT *Km^R^* FRT | [5] |
| pKD46 | rep_pSC101_^ts^ *Amp^R^* P_araBAD_-γβexo | [5] |
| pUHE-21 | rep_pMB1_ *lacI^q^* *Amp^R^* vector control | [8] |
| pUHE-UgtS | rep_pMB1_ *lacI^q^* *Amp^R^* P_lac_-*ugtS* | This study |
| pUHE-UgtS_Typhi_ | rep_pMB1_ *lacI^q^* *Amp^R^* P_lac_-*ugtS_Typhi_* | This study |
| pSLC-242 | rep_R6Kγ_ *Amp^R^* FRT-(*Cm^R^* P*_rhaB_*-*relE*)-FRT | [9] |
| pXG10sf | rep _pSC101*_ *Cm^R^* P_LtetO_-*lacZ-gfp* | [10] |
| pXG10sf-*ugtS-182* | rep _pSC101*_ *Cm^R^* P_LtetO_-*ugtS-182-gfp* | This study |
| pXG10sf-*ugtSmutAUG-182* | rep _pSC101*_ *Cm^R^* P_LtetO_-*ugtSmutAUG-182-gfp* | This study |
| pXG10sf-*ugtS-171* | rep _pSC101*_ *Cm^R^* P_LtetO_-*ugtS-171-gfp* | This study |

**References**

1. Hanahan D. Studies on transformation of Escherichia coli with plasmids. J Mol Biol. 1983;166(4):557-80. Epub 1983/06/05. PubMed PMID: 6345791.

2. Fields PI, Swanson RV, Haidaris CG, Heffron F. Mutants of Salmonella typhimurium that cannot survive within the macrophage are avirulent. Proc Natl Acad Sci U S A. 1986;83(14):5189-93. Epub 1986/07/01. PubMed PMID: 3523484; PubMed Central PMCID: PMCPMC323916.

3. Fields PI, Groisman EA, Heffron F. A Salmonella locus that controls resistance to microbicidal proteins from phagocytic cells. Science. 1989;243(4894 Pt 1):1059-62. Epub 1989/02/24. doi: 10.1126/science.2646710. PubMed PMID: 2646710.

4. Choi J, Groisman EA. Activation of master virulence regulator PhoP in acidic pH requires the Salmonella-specific protein UgtL. Sci Signal. 2017;10(494). Epub 2017/08/31. doi: 10.1126/scisignal.aan6284. PubMed PMID: 28851823; PubMed Central PMCID: PMCPMC5966036.

5. Datsenko KA, Wanner BL. One-step inactivation of chromosomal genes in Escherichia coli K-12 using PCR products. Proc Natl Acad Sci U S A. 2000;97(12):6640-5. Epub 2000/06/01. doi: 10.1073/pnas.120163297. PubMed PMID: 10829079; PubMed Central PMCID: PMCPMC18686.

6. McKenzie GJ, Craig NL. Fast, easy and efficient: site-specific insertion of transgenes into enterobacterial chromosomes using Tn7 without need for selection of the insertion event. BMC Microbiol. 2006;6:39. Epub 2006/05/02. doi: 10.1186/1471-2180-6-39. PubMed PMID: 16646962; PubMed Central PMCID: PMCPMC1475584.

7. Zeghouf M, Li J, Butland G, Borkowska A, Canadien V, Richards D, et al. Sequential Peptide Affinity (SPA) system for the identification of mammalian and bacterial protein complexes. J Proteome Res. 2004;3(3):463-8. Epub 2004/07/16. doi: 10.1021/pr034084x. PubMed PMID: 15253427.

8. Soncini FC, Vescovi EG, Groisman EA. Transcriptional autoregulation of the Salmonella typhimurium phoPQ operon. J Bacteriol. 1995;177(15):4364-71. Epub 1995/08/01. PubMed PMID: 7543474; PubMed Central PMCID: PMCPMC177185.

9. Khetrapal V, Mehershahi K, Rafee S, Chen S, Lim CL, Chen SL. A set of powerful negative selection systems for unmodified Enterobacteriaceae. Nucleic Acids Res. 2015;43(13):e83. Epub 2015/03/25. doi: 10.1093/nar/gkv248. PubMed PMID: 25800749; PubMed Central PMCID: PMCPMC4513841.

10. Corcoran CP, Podkaminski D, Papenfort K, Urban JH, Hinton JC, Vogel J. Superfolder GFP reporters validate diverse new mRNA targets of the classic porin regulator, MicF RNA. Mol Microbiol. 2012;84(3):428-45. Epub 2012/03/31. doi: 10.1111/j.1365-2958.2012.08031.x. PubMed PMID: 22458297.
