# Supplementary material for: Differential synthesis of novel small protein times Salmonella virulence program
Source: PLoS Genet. 2022 Mar 4;18(3):e1010074. doi: 10.1371/journal.pgen.1010074 (PMC8896665; doi:10.1371/journal.pgen.1010074)
Supplement: S2 Table — (DOCX) [file pgen.1010074.s005.docx]

**S2 Table.** Oligonucleotides sequences used in this study

| **Name** | **Sequence (5′ -> 3′)** | **Purpose** | **Source** |
| --- | --- | --- | --- |
| 6627 | ggcgaccgtagtaatatcgacaa | *pcgL* qRT-PCR | This study |
| 6628 | ctttcctcctgttcagcctgtt | *pcgL* qRT-PCR | This study |
| 6962 | gcaggagtaatatgttggacagtcac | *mgtC* qRT-PCR | This study |
| 6963 | gggagattgctgcccacc | *mgtC* qRT-PCR | This study |
| 6964 | aaaagattaaatcggagcggga | *pagC* qRT-PCR | This study |
| 6965 | tgacgctccatccgcaata | *pagC* qRT-PCR | This study |
| 14514 | GGTTAAGAAATCGCATTATGTCAAAA | *pmrD* qRT-PCR | This study |
| 14515 | CGAACCGCCGCTATCG | *pmrD* qRT-PCR | This study |
| 16655 | ATTCTCACATCATCATGTACTATGAGTAATGATTAATTACGCACTATATTATTTTTAGAGGTGTAGGCTGGAGCTGCTTCGAAGTTCCTATACTTTCTAG | Generation of *Plac1-6_-12ugtL*::Cm^R^ strain | This study |
| 16658 | CAGGATGCTGTCTTTTCGTGAATTTCACCATCTGATTTCTTCATTTTGAGCCTCCTTCCACACAACATAAACATAAAAAGCTTAAAGTGTAAAGATATGAATATCCTCCTTAGTTCCTATTCCGAAGTTCCTAT | Generation of *Plac1-6_-12ugtL*::Cm^R^ strain | This study |
| 16686 | GGTTCAGCGCTTGTCCAAAAAATTTGGGGCACAGGATGTTTCTTCACGCCCGGACTACAAGGACGACGATGACAAGTGATGTAGGCTGGAGCTGCTTCG | Generation of *Plac1-6_-12ugtL-FLAG*::Km^R^ strain | This study |
| 16687 | AGATAATTCGGTCTGCTGGTCATAGCCATTATTCAGTAAGACCGCAGGTTGCAGCGGCGGAATATGAATATCCTCCTTAGTTC | Generation of *Plac1-6_-12ugtL-FLAG*::Km^R^ strain | This study |
| 16864 | TTACTGAGAAGTTAAATCATGCTTCGCTTGATTTATGGCATATCCTTAAGGACTACAAGGACGACGATGACAAGTGATGTAGGCTGGAGCTGCTTCG | Generation of *Plac1-6_-12ugtL_1-89truncation_-FLAG::Km^R^* strain | This study |
| 17857 | GTTTTATGCATATTATTAGACTAACAACAATGAGATGTTTAGCGGTAG | Cloning of *ugtSmutSD* from -182 position relative to *ugtL* ATG start codon | This study |
| W856 | TGTTTAGCGGTAGGGCAGAAG | *ugtL* qRT-PCR | This study |
| W857 | CCGTCAGCTAATCGTTACAACAA | *ugtL* qRT-PCR | This study |
| W912 | caattgtgagcggataacaatttc | Sequencing of pUHE-21 inserts | This study |
| W913 | aatccagatggagttctgagg | Sequencing of pUHE-21 inserts | This study |
| W1332 | TGGGATATATCAACGGTGGT | Sequencing of pXG10sf inserts | This study |
| W1333 | CCGTATGTAGCATCACCTTC | Sequencing of pXG10sf inserts | This study |
| W1883 | CCAGCAGCCGCGGTAAT | *rrs* qRT-PCR | This study |
| W1884 | TTTACGCCCAGTAATTCCGATT | *rrs* qRT-PCR | This study |
| W3364 | GTTTTATGCATAACAACAATGAGATGTTTAG | Cloning of *ugtS* from -171 position relative to *ugtL* ATG start codon | This study |
| W3503 | GTTTTATGCATATTATTAGGCTAACAACAATGAG | Cloning of *ugtS* from -182 position relative to *ugtL* ATG start codon | This study |
| W3558 | CTCACATCATCATGTACTATGAG | DNA template generation for *ugtL*/*ugtS* primer extension sequencing ladder and confirmation of *Plac1-6_-12ugtL*::Cm^R^ insertion | This study |
| W3559 | GATGCTGTCTTTTCGTGA | DNA template generation for *ugtL*/*ugtS* primer extension sequencing ladder and confirmation of *Plac1-6_-12ugtL*::Cm^R^ insertion | This study |
| W3560 | CATTTTGAGCCTCCTCGCAG | Sequencing primer for *ugtSmutAUG* allele insertion confirmation | This study |
| W3621 | ATTCTCACATCATCATGTAC | Sequencing primer for *ugtSmutAUG* allele insertion confirmation | This study |
| W3781 | TTGTCTTATAAGAAGTTAAACTAAAAGTATTATTAGGCTAgtgtaggctggagctgcttc | Generation of *ugtSmutAUG* strain | This study |
| W3782 | TATATCATAAGCACTATCAGTATTGGCCTTCTGCCCTACCcatatgaatatcctccttag | Generation of *ugtSmutAUG* strain | This study |
| W3783 | TTGTCTTATAAGAAGTTAAACTAAAAGTATTATTAGGCTAACAACATAGAGATGTTTAGCGGTAGGGCAGAAGGCCAATACTGATAGTGCTTATGATATA | Generation of *ugtSmutAUG* strain | This study |
| W3784 | TATATCATAAGCACTATCAGTATTGGCCTTCTGCCCTACCGCTAAACATCTCTATGTTGTTAGCCTAATAATACTTTTAGTTTAACTTCTTATAAGACAA | Generation of *ugtSmutAUG* strain | This study |
| W3785 | GTTTTATGCATATTATTAGGCTAACAACATAGAGATGTTTAGCGGTAG | Cloning of *ugtSmutAUG* from -182 position relative to *ugtL* ATG start codon | This study |
| W3786 | GTTTTTGCTAGCATCGTTACAACAAATATAATTAAGAC | Cloning of *ugtS* | This study |
| W3859 | GCATCGAATTCATTATTAGGCTAACAACAATGAG | Cloning of *ugtS* | This study |
| W3860 | GCATCAAGCTTAAACAAAGCCGTCAGCTAATC | Cloning of *ugtS* | This study |
| W3871 | GTCCAAAAAATTTGGGGCACAGGATGTTTCTTCACGCCCGGACTACAAGGACGACGATGACAAGTAACATATGAATATCCTCCTTA | Generation of *ugtL-FLAG::Cm^R^* strain | This study |
| W3872 | ATAGCCATTATTCAGTAAGACCGCAGGTTGCAGCGGCGGAGTGTAGGCTGGAGCTGCTTC | Generation of *ugtL-FLAG::Cm^R^* strain | This study |
| W3873 | CCTTAAGTTATGCCTATG | *ugtL-FLAG::Cm^R^* verification | This study |
| W3874 | CTCGAACCGAGATAATTCG | *ugtL-FLAG::Cm^R^* verification | This study |
| W3999 | ACTCTTTAGTTTTTGTCTTAATTATATTTGTTGTAACGATTCCATGGAAAAGAGAAG | Generation of *ugtS-SPA::Km^R^* strain | This study |
| W4000 | TTTTATAATTTTATCGCCCAACTGGAAACAAAGCCGTCAGCATATGAATATCCTCCTTAG | Generation of *ugtS-SPA::Km^R^* strain | This study |
| W4001 | GAAGGCCAATACTGATAGTG | Sequencing primer for *ugtS-SPA* allele insertion confirmation | This study |
| W4002 | CACCATCTGATTTCTTCAT | Sequencing primer for *ugtS-SPA* allele insertion confirmation | This study |
| W4055 | CACTATCAGTATTGGCCTTC | *ugtL* primer extension (-122 to -141 region relative to *ugtL* ATG start codon) | This study |
| W4094 | CATAGCCATTATTCAGTAAGACCGCAGGTTGCAGCGGCGGgtgtaggctggagctgcttc | *ugtL* inactivation | This study |
| W4095 | GTAGCTTTAGTCATGCCCACGCCTC | Δ*ugtLORF*::Cm^R^ verification | This study |
| W4096 | CACTGCAGTTGTGCAAACAC | Δ*ugtLORF*::Cm^R^ verification | This study |
| W4220 | GCGAATTAATACGACTCACTATAGGGCTTAAGTATAAGGAGGAAAAAATATGAGATGTTTAGCGGTAGGGCAGAAG | Generation of *ugtS-HA* template | This study |
| W4222 | GCGAATTAATACGACTCACTATAGGGCTTAAGTATAAGGAGGAAAAAATATGAAGAAATCAGATGGTGAAATTCACGAAAAGAC | Generation of *ugtL-FLAG* template | This study |
| W4302 | AAACCCCTCCGTTTAGAGAGGGGTTATGCTAGTCATTACTTGTCATCGTCGTCCTTGTAGTCCGGGCGTGAAGAAACATCCTGTGCCCCAAATTTTTTGGACAAGCGCTGAACCTGCCGT | Generation of *ugtL-FLAG* template | This study |
| W4319 | GCGAATTAATACGACTCACTATAGGGCTTAAGTATAAGGAGGAAAAAATATGAATAAATTTGCTCGCCATTTTCTGCCGCTGTC | Generation of *phoQ-FLAG* template | This study |
| W4320 | AAACCCCTCCGTTTAGAGAGGGGTTATGCTAGTTACTTGTCATCGTCGTCCTTGTAGTCTTCCTCTTTCTGTGTGGGATGCTGTCGGCCAA | Generation of *phoQ-FLAG* template | This study |
| W4463 | TTGGGCGATAAAATTATAAAAACCTGCGAGGAGGCTCAAAcatatgaatatcctccttag | *ugtL* inactivation | This study |
| W4466 | AGATCATGACATCGACTACAAGGATGACGATGACAAGTAGgtgtaggctggagctgcttc | Generation of *ugtS-SPA* strain | This study |
| W4467 | AGATCATGACATCGACTACAAGGATGACGATGACAAGTAGCTGACGGCTTTGTTTCCAGTTGGGCGATAAAATTATAAAA | Generation of *ugtS-SPA* strain | This study |
| W4468 | TTTTATAATTTTATCGCCCAACTGGAAACAAAGCCGTCAGCTACTTGTCATCGTCATCCTTGTAGTCGATGTCATGATCT | Generation of *ugtS-SPA* strain | This study |
| W4469 | GACAAAAACTAAAGAGTAAG | *ugtL* primer extension (-105 to -86 region relative to *ugtL* ATG start codon) | This study |
| W4634 | CAGTTCTCGAGATCGGTTTGCCTATCGGTGAAACAC | Cloning of *ugtS-SPA* | This study |
| W4635 | GCATCCTCGAGCAGCTACTTGTCATCGTCAT | Cloning of *ugtS-SPA* | This study |
| W4636 | gatgctggtggcgaaactgtc | Sequencing primer for *ugtS-SPA* allele insertion in the *att*Tn7 attachment site | This study |
| W4637 | gaagagtggaacgtcggtac | Sequencing primer for *ugtS-SPA* allele insertion in the *att*Tn7 attachment site | This study |
| W4638 | ctagtattaccctgttatccctag | Sequencing of pGRG25 inserts | This study |
| W4639 | gaagtgcaaattgcccgtcg | Sequencing of pGRG25 inserts | This study |
| W4804 | AAACCCCTCCGTTTAGAGAGGGGTTATGCTAGCTAAGCGTAATCTGGAACATCGTATGGGTAATCGTTACAACAAATATAATTAAGACA | Generation of *ugtS-HA* template | This study |
| W4932 | GCGAATTAATACGACTCACTATAGGGCTTAAGTATAAGGAGGAAAAAATATGTCACAGGTTACTGAAAATAATGTTAATG | Generation of *dppC-FLAG* template | This study |
| W4933 | AAACCCCTCCGTTTAGAGAGGGGTTATGCTAGTTATTACTTGTCATCGTCGTCCTTGTAGTCCTGCTTCAGTTTGGGATCGAGCGCGTCACG | Generation of *dppC-FLAG* template | This study |
